# Gene-specific response to muscle specific kinase agonist antibody in the treatment of congenital myasthenic syndromes

**DOI:** 10.1093/braincomms/fcag115

**Published:** 2026-05-14

**Authors:** Kelly Ho, Ofosu Adjei-Afriyie, Ricardo Carmona-Martinez, Rohan Ray, Daniel O’Neil, Joshua Zeldin, Julien Oury, Lieselot De Clercq, Steven J Burden, Bernhardt Vankerckhoven, Roeland Vanhauwaert, Sally Spendiff, Hanns Lochmüller

**Affiliations:** Children’s Hospital of Eastern Ontario Research Institute, Ottawa, ON K1H 5B2, Canada; Department of Cellular and Molecular Medicine, University of Ottawa, Ottawa, ON K1H 8L1, Canada; Children’s Hospital of Eastern Ontario Research Institute, Ottawa, ON K1H 5B2, Canada; Department of Cellular and Molecular Medicine, University of Ottawa, Ottawa, ON K1H 8L1, Canada; Children’s Hospital of Eastern Ontario Research Institute, Ottawa, ON K1H 5B2, Canada; Children’s Hospital of Eastern Ontario Research Institute, Ottawa, ON K1H 5B2, Canada; Department of Cellular and Molecular Medicine, University of Ottawa, Ottawa, ON K1H 8L1, Canada; Children’s Hospital of Eastern Ontario Research Institute, Ottawa, ON K1H 5B2, Canada; Children’s Hospital of Eastern Ontario Research Institute, Ottawa, ON K1H 5B2, Canada; Department of Cellular and Molecular Medicine, University of Ottawa, Ottawa, ON K1H 8L1, Canada; Tevard Biosciences, Cambridge, MA 02139, USA; argenx, Zwijnaarde, 9052 Ghent, Belgium; Department of Neurology, Massachusetts General Hospital, Harvard University, Boston, MA 02114, USA; argenx, Zwijnaarde, 9052 Ghent, Belgium; argenx, Zwijnaarde, 9052 Ghent, Belgium; Children’s Hospital of Eastern Ontario Research Institute, Ottawa, ON K1H 5B2, Canada; Children’s Hospital of Eastern Ontario Research Institute, Ottawa, ON K1H 5B2, Canada; Department of Cellular and Molecular Medicine, University of Ottawa, Ottawa, ON K1H 8L1, Canada; Division of Neurology, Department of Medicine, The Ottawa Hospital, Ottawa, ON K1Y 1J8, Canada; Brain and Mind Research Institute, University of Ottawa, Ottawa, ON K1H 8L1, Canada; Department of Neuropediatrics and Muscle Disorders, Medical Center—University of Freiburg, 79085 Freiburg, Baden-Württemberg, Germany; Centro Nacional de Análisis Genómico, 08028 Barcelona, Catalonia, Spain

**Keywords:** neuromuscular disease, neuromuscular junction, congenital myasthenic syndromes, preclinical trial

## Abstract

Congenital myasthenic syndromes (CMS) are a group of rare disorders characterized by fatigable muscle weakness and caused by impaired neuromuscular junction (NMJ) function. CMS symptoms are highly variable, but it can be detrimental and lead to death. There are over 40 different genetic subtypes, including *AGRN-*CMS and *COLQ-*CMS. *AGRN* encodes for neuralagrin, which is released from the nerve terminal and triggers muscle-specific kinase phosphorylation (pMuSK). pMuSK is essential for NMJ development and maintenance, thus agrin deficiency causes NMJ impairment. *COLQ* encodes for collagenous subunit Q (ColQ), which anchors acetylcholinesterase and stabilizes MuSK. As a result, COLQ deficiency results in NMJ degeneration from prolonged transmission signals and decreased pMuSK. Current treatments for *AGRN-*CMS and *COLQ-*CMS are limited, highlighting the importance of finding more efficient therapies. Recently, a MuSK agonist antibody (ARGX-119) with high affinity for the Frizzled-like domain showed remarkable rescue of a *Dok7*-CMS mouse model. We hypothesized a derivative antibody of ARGX-119 (3B2) could benefit *Agrn-* and *ColQ-*CMS mouse models. *Agrn-*CMS mice were treated at postnatal day 5 (P5), P15 and P35, and *ColQ-*CMS mice were treated weekly from P22 to P57. In *Agrn-*CMS mice, 3B2 treatment rescued survival, bodyweight, fibre type switching and pMuSK levels, and improved forelimb grip strength and NMJ morphology. In *ColQ-*CMS mice, 3B2 treatment was unable to rescue deficits observed. Our findings suggest that MuSK agonists may benefit patients with *AGRN*-CMS, which should be tested in clinical trials. Our study emphasizes that effective CMS treatment is gene-dependent and relies on an accurate genetic diagnosis.

## Teaser

Ho, Adrie-Afriyie *et al.* investigate the therapeutic potential of muscle-specific kinase antibody treatment in *AGRN* and *COLQ* genetic subtypes of congenital myasthenic syndromes, a rare neuromuscular disease. They report that the treatment had remarkable improvements in the *AGRN* mouse model, but it was ineffective in the *COLQ* mouse model.

## Graphical Abstract

**Graphical Abstract:**
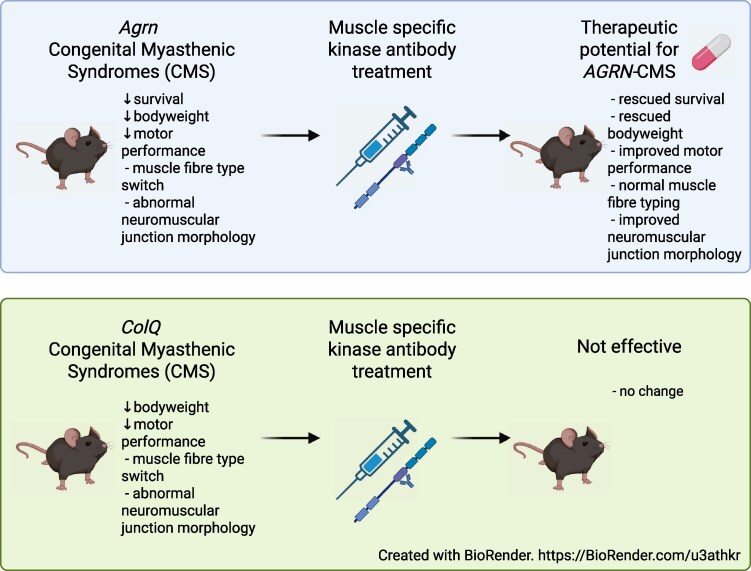

## Introduction

In mice and humans, synaptic transmission at the neuromuscular junction (NMJ) involves the release of acetylcholine (ACh) from synaptic vesicles into the synaptic cleft. ACh crosses the synaptic cleft and binds to ACh receptors (AChRs) in the muscle fibre membrane. The resulting opening of AChR channels depolarizes the muscle, initiating an action potential, which leads to muscle fibre contraction. This process is rapidly terminated by acetylcholinesterase (AChE), which hydrolyzes ACh and is anchored to the basal lamina by a collagen tail (COLQ).^1^

Efficient synaptic transmission requires clustering of AChRs at the crests and upper portions of postjunctional folds, which are directly opposite to transmitter release sites, active zones, in motor nerve terminals.^1^ The formation and maintenance of these pre- and postsynaptic specializations relies on the AGRIN/LRP4/MuSK pathway, reviewed in Ohno *et al*.^2^ Neural agrin is released from motor nerve terminals and binds to low density lipoprotein receptor 4 (LRP4) in the postsynaptic membrane. Agrin binding promotes association between LRP4 and muscle-specific kinase (MuSK) and stimulates MuSK tyrosine phosphorylation, which is essential for AChR clustering.^3,4^ Phosphorylated MuSK (pMuSK) recruits downstream tyrosine kinase 7 (DOK7), which further aids in the phosphorylation of MuSK.^5,6^ Activated MuSK leads to tyrosine phosphorylation of AChRs, which augments the association of AChRs to receptor-associated protein of the synapse (rapsyn) and assists in anchoring AChRs to the cytoskeleton.^7-9^

Congenital myasthenic syndromes (CMS) are a diverse set of genetic neuromuscular conditions caused by impaired synaptic transmission at the NMJ. Clinically, CMS commonly presents within the first 2 years of life and is characterized by abnormal fatigable muscle weakness. The ocular and limb muscles are particularly affected, and the disease can be severe and even fatal with respiratory failure. Currently, mutations in 40 genes have been shown to cause CMS.^10^  *DOK7* is one of the most frequently mutated genes in CMS, and patients with *DOK7*-CMS can suffer debilitating symptoms.^11,12^ The most common disease-causing *DOK7* mutation causes truncation of the protein, leading to reduced MuSK tyrosine phosphorylation.^13^ MuSK agonist antibodies (X17 and ARGX-119) provide remarkable benefit in a *DOK7-*CMS mouse model,^13,14^ raising the possibility that treatment with a MuSK agonist antibody might provide similar benefit for other forms of CMS caused, at least in part, by reduced MuSK phosphorylation.

To investigate this hypothesis, we studied a mouse model of *AGRN-*CMS that has an N-ethyl N-nitrosourea induced point mutation in the *nmf380* locus of mouse *Agrn* resulting in partial loss of function of the allele.^15^ The *nmf380* locus is part of the SEA (Sperm protein, Enterokinase and Agrin) domain, which was identified to have important NMJ functions. More recently, an *AGRN-*CMS case due to homozygous mutations in the SEA domain was reported.^16^ Mice homozygous for this mutation are runted, display poor motor control, and usually die within a few weeks after birth.^15,17^ We also studied a second CMS mouse model lacking ColQ.^18-21^ Triple helical ColQ binds to AChE to assist in anchoring AChE to the synaptic basal lamina. Although loss of function mutations in *ACHE* are incompatible with life, many CMS cases are caused by hypomorphic mutations in *COLQ.*^2,22,23^  *ColQ* knockout (*ColQ*^−/−^) mice, which lack basal lamina-attached AChE, are runted, exhibit muscle weakness, and display structural abnormalities of the NMJ. *ColQ^−/−^* mice were initially reported to rarely survive into adulthood (10–20%),^21^ but more recent studies reported longer survival of *ColQ^−/−^* mice up to 9 weeks of age.^24^ Although ColQ is not a component of the agrin/LRP4/MuSK pathway, it is reported to bind the Ig-like 1 and Frizzled-like (Fz-like) domains of MuSK^25^ and to stabilize MuSK at the muscle membrane.^18-20,25^ As such, we considered the possibility that boosting MuSK phosphorylation might ameliorate disease symptoms in *ColQ^−/−^* mice.

MuSK agonist antibody (ARGX-119) treatment is currently undergoing clinical trials for *DOK7*-CMS (NCT06436742), so we hypothesized that other forms of CMS may benefit from this treatment strategy. Our results demonstrated that 3B2, a derivative antibody of ARGX-119 with similar MuSK binding properties and MuSK activation, rescued many of the cellular and phenotypic characteristics of *Agrn^nmf380^* mice, but was ineffective in rescuing *ColQ^−/−^* mice. These findings suggest a possible therapy for *AGRN*-CMS and highlight the importance of a correct genetic diagnosis for the treatment of CMS patients.

## Materials and methods

### Sex as a biological variable

CMS is inherited in a Mendelian fashion and thus, there are no known sex differences in prevalence, age of onset, disease severity or treatment response. A retrospective study of 235 adult CMS patients reported that 16.2% of female patients had worsened symptoms during menstruation and 32.4% of female pregnant patients had worsened symptoms during pregnancy.^26^ This suggests that hormonal changes can impact CMS symptoms. Therefore, for survival, bodyweight, and motor performance, we ensured equal sexes in each group by including the first mice that survived until the end of the study from each sex and excluding additional mice that made the sex numbers unequal. Furthermore, for bodyweight and motor behavioural testing, we analysed males and females separately to ensure no sex differences before merging the data.

### Animal husbandry

*Agrn^nmf380^* mice were obtained from the Burgess lab^15^ and *ColQ*^−/−^ mice^21^ were provided through kind donation by the Krejci laboratory (Université Paris Descartes). These mice were rederived at the University of Ottawa by the animal care and veterinary service department (ACVS) transgenic core under breeding protocol 3089. Animals were housed under 12-hour light/dark cycles and had *ad libitum* access to standard chow, water and soaked diet on the cage floor. Experimental mice were housed at the same cage locations to minimize potential confounders. Animals were weighed at least 3 times per week, and any animal found to have lost >17% of bodyweight or showing a severe phenotype as specified on our ‘in house’ monitoring sheet was humanely culled. All animal procedures were performed following the approval of the University of Ottawa Animal Care Committee (experimental protocol 3120) and complied with the guidelines of the Canadian Council on Animal Care and the Animals for Research Act. The experimental protocol was prepared before the study commenced and is contained within a signed contract between the Children’s Hospital of Eastern Ontario Research Institute and argenx. Animal sample sizes were determined based on power analysis using grip strength and NMJ morphology analysis data from previous studies with the same mouse models,^17,24^ and litters were assigned at random to control or experimental conditions. All animals were named by an identification number that did not indicate which treatment group they were in. However, due to the phenotype of the mice and the remarkable response to treatment in the *Agrn^nmf380^* mice, it is likely that the mice were identifiable through their phenotype.

### Muscle strength assessments

Mice underwent strength assessments according to standard operating protocols.^27^ In the hindlimb suspension test, each mouse was suspended over the edge of a 50 mL Falcon tube containing bedding. The mouse was assessed for the time to fall, relative positioning of the hindlimbs (hindlimb suspension score), and number of attempts the mouse made at pulling itself out of the tube. The hindlimb suspension score was determined as follows: score of 4 indicates normal hindlimb separation (feet stance is wider than width of body) with tail raised, score of 3 indicates that weakness is apparent, with hindlimbs closer together but not touching (feet stance is about the width of body), score of 2 indicates that hindlimbs are close together and often touching, score of 1 indicates that the hindlimbs are almost always in a clasped position with the tail raised, and a score of 0 indicates that there is constant clasping of the hindlimbs with the tail lowered, or failure to hold onto the Falcon tube at all. Each mouse underwent two trials with 5 min of rest between attempts. To assess forelimb grip strength, the mouse was held by the tail and allowed to grab the grid with its forepaws before it was pulled away horizontally. After at least 10 min of rest, the mice were assessed for hindlimb grip strength, whereby they were held by the scruff of the neck and tail and presented to the grid at a 45° angle to allow only the hindlimbs to grip before being pulled horizontally. For both forelimb and hindlimb grip strength, each mouse was tested three times with 30 s of rest and the results averaged. To normalize for bodyweight, forelimb and hindlimb grip strength ratio were determined by dividing grip strength in Newtons by bodyweight in grams. For the inverted screen test, the mouse was acclimatized to the grid for 1 min before the grid was inverted to suspend the mouse over a padded container. Each mouse was tested three times with a 10-minute rest period between each trial and the results averaged. To normalize for bodyweight, holding impulse was calculated by multiplying time to fall by mouse bodyweight in grams. Mice were acclimatized to the behavioural room for at least 30 min before grip strength and inverted screen tests were performed.

### Tissue collection

Muscle for labelling was mounted in Optimum Cutting Temperature compound (OCT) and frozen in pre-cooled isopentane before being stored at −80°C. Muscle was cryosectioned on a Leica CM1860 at 10 µm and mounted on slides. Before labelling, slides were thawed at room temperature (RT) for 20 min. Muscle for protein analysis was snap frozen in liquid nitrogen and stored at −80°C.

### Muscle histology

#### Haematoxylin and eosin (H & E)

Sections were placed in tap water for 30 s, then into haematoxylin for 2 min. They were washed in tap water and placed in eosin for 30 s. They were washed in tap water before dehydrating through a graded ethanol series that finished with two clears using xylene. Sections were mounted with a cover slip and DPX Mountant for histology (Millipore Sigma). Tiled images were taken on a Zeiss Axio Imager at 20× magnification. Fibre area was determined using an automated system.^28^

#### Myosin heavy chain (MHC) type and muscle fibre size

Sections for labelling of MHCs (fibre type) and laminin were washed in phosphate buffered saline (PBS), then blocked in 10% normal goat serum (NGS) for one hour at RT. Primary antibodies were obtained from Developmental Studies Hybridoma Bank; mouse anti-MHC1 IgG2b (BA-F8, 1:25), mouse anti-MHC2a IgG1 (SC-71, 1:200), and mouse anti-MHC2b (BF-F3, 1:200). These were applied to one section and on a second serial section, mouse anti-MHC2x IgGM (6H1, 1:20) was labelled. Both sections were also stained with rabbit anti-laminin IgG (1:750, Sigma, L9393). Muscles were incubated for 1 h at RT and then washed in PBS. Secondary antibodies from ThermoFisherScientific were applied for 1 h at RT: Alexa Fluor 350 IgG2b (y2b) goat anti-mouse (A-21140, 1:500), Alexa Fluor 594 IgG1 (y1) goat anti-mouse (A-21125, 1:100), Alexa Fluor 488 IgM goat anti-mouse (A-21042, 1:500), and Alexa Fluor 488 IgG goat anti-rabbit (A-11008, 1:500). Following this, sections were given final washes and mounted using Vectashield hardset mounting medium (Vector Laboratories). Tiled images of the muscle section were captured on a Zeiss Axio Imager M2 microscope. Analysis was performed blinded with the Zeiss Zen software. In the software, fibres (>20 µm) were identified using laminin and fibre MHC was determined using the histogram function for each channel.

#### NMJ labelling and analysis

Soleus muscles were washed in ice-cold PBS for 2 × 10 min and separated out into small bundles using tweezers under a stereomicroscope. They were fixed overnight at 4°C in 2% paraformaldehyde (PFA). The following morning, they were washed 2 × 1 h with cold PBS. They were treated for 10 min with Analar Ethanol followed by 10 min with Analar Methanol, both at −20°C. Tissues were incubated with blocking/permeabilization solution (5% horse serum (HS), 5% bovine serum albumin (BSA), 2% Triton X-100 in PBS) for 4 h at RT with gentle agitation. Samples were incubated with antibodies, diluted in blocking buffer (5% HS, 5% BSA), against neurofilament (mouse monoclonal IgG1, Cell Signalling, 1:100) and synaptophysin (rabbit polyclonal, ThermoFisherScientific, 1:100) overnight at 4°C with agitation and for a further 2 h at RT the next morning. Muscles were washed in blocking buffer 4 × 1 h at RT. They were incubated with Alexa 488-Conjugated α-Bungarotoxin (α-BTX) (ThermoFisherScientific, 1:250), and Alexa Fluor 594 goat anti-mouse IgG1 (ThermoFisherScientific, 1:200) for 4 h at RT or overnight at 4°C with agitation. Samples were washed 4 × 1 h in PBS and mounted using Vectashield hardset mounting medium. Images were captured using Olympus FV1000c scanning confocal microscope with FV1000 application software (FV10-ASW) software. Z-stack images at 1 μm intervals were acquired with a 63× oil immersion objective. Analysis was performed blinded according to the NMJ_Morph protocol.^29^ An average of 9.93 NMJs were analysed per mouse in the *Agrn-*CMS study, and an average of 20.72 NMJs were analysed per mouse in the *ColQ-*CMS study.

### Immunostaining of MuSK and pMuSK cryosections

The tissue was outlined with a PAP Pen (Sigma Z672548), dried for 30 min and rehydrated with PBS for 10 min. The tissue was fixed in 2% PFA containing a Phosphatase Inhibitor Cocktail tablet (Roche 4906845001) for 10 min at RT. PBS washes were performed, then the tissue was permeabilized with 0.1% Triton-X in PBS for 10 min at RT, followed by washing. The tissue was then incubated in blocking solution (10% goat serum + 1% BSA in PBS) for 1 h. Primary antibodies, MuSK (Life Technologies PA1–1741, 1:50) and pMuSK Y755 (Abcam ab192583, 1:100) were applied overnight at 4°C in a humidified chamber. After washing, the sections were incubated with Alexa Fluor™ 488 α-BTX conjugate (B13422, 1:100) and Alexa Fluor 594 Goat anti-Rabbit (A-11012, 1:100) for 2 h at RT. Following PBS washes, slides were mounted with Vectashield Mounting Medium (H-1000-10), allowed to dry, and sealed with clear nail polish. Images were captured using a 40× objective on the Zeiss Axio Imager M2.

Quantification of synaptic MuSK and pMuSK, and extrasynaptic MuSK was conducted blinded with Zeiss imaging software Zen Blue 3.2. A custom set of instructions in Zen Blue 3.2 were generated to semi-automatically trace α-BTX staining and provide measurements, including α-BTX area (µm^2^) and mean intensity values for channel AF594 (MuSK and pMuSK). With the automatic segmentation feature in Zen Blue 3.2, the area where the intensity of the channel traces the NMJs is selected in the histogram. With the selected areas, the functions ‘Fill Holes,’ ‘Erode function’ and ‘Morphology’ are selected to generate the area and intensity mean value of the channel. Background noise from AF594 channel value was calculated and subtracted from mean intensity values. Integrated density was calculated by multiplying α-BTX area (µm^2^) with mean intensity values of channel AF594. Quantification of extrasynaptic MuSK was performed by taking the integrated density of total MuSK (multiplication of area (µm^2^) with mean intensity values of channel AF594), subtracting background noise from channel AF594. This total MuSK value was subtracted by the synaptic MuSK values (calculated as described above) from the same slide. An outlier test for all MuSK or pMuSK integrated density values per mouse was performed and the averages of these values were plotted.

### Quantification of MuSK and pMuSK in muscle lysate through Western blot

Tibialis anterior (TA) muscles in ice-cold lysis buffer (RIPA Buffer, protease inhibitor, PhosSTOP) were homogenized using a Qiagen Tissue Lyser at 30 Hz for 3 cycles of 90 s, with 2 min of cooling on ice between cycles. The samples were rotated end-over-end at 4°C overnight. The samples were sonicated using a Sonics Vibracell Ultrasonic Processor at 20% power for 3 cycles of 3 s, with 2 min of cooling on ice between cycles. The samples were centrifuged at 16 000 g for 30 min at 4°C and the supernatant was collected. The protein concentration was measured using a DC protein assay (BioRad 5000112). The samples were denatured by adding Laemmli buffer (BioRad 1610747) and heated.

40 µg of protein was loaded on an 8% 1.5 M Tris-HCl gel and electrophoresed. The proteins were transferred to a pre-activated polyvinylidene difluoride (PVDF) membrane (BioRad 1620177). The membrane was blocked with EveryBlot blocking buffer (BioRad 12010020) for 15 min at RT. The membrane was incubated with a 1:400 dilution of anti-MuSK (phospho Y755) antibody (abcam ab192583) or 1:100 dilution of MuSK Antibody (1-YD2) (Santa Cruz sc-134398) in EveryBlot blocking buffer for 40 h (pMuSK) or overnight (MuSK) at 4°C. The membrane was washed with tris-buffered saline 0.1% tween (TBST). The membrane was incubated with a 1:3000 dilution of goat anti-rabbit IgG secondary antibody, HRP (ThermoFisherScientific 31460) in EveryBlot blocking buffer for 1 h at RT for pMuSK detection. The membrane was incubated with a 1:2000 dilution of goat anti-mouse IgG secondary antibody, HRP (ThermoFisherScientific 31430) in EveryBlot blocking buffer for 1 h at RT for MuSK detection. After washing, the membrane was treated with Clarity Max Western ECL substrate (BioRad 1705062) for five minutes and imaged using a BioRad ChemiDoc imaging system. The membrane was washed and incubated with a 1:1000 dilution of vinculin antibody 7F9 (Santa Cruz sc-73614) in 5% milk/TBST for 1 h at RT. After washing, the membrane was incubated with a 1:3000 dilution of goat anti-mouse IgG secondary antibody, HRP (ThermoFisherScientific 31430) in 5% milk/TBST for 1 h at RT. After washing, bands were detected using Clarity Western ECL substrate (BioRad 1705061) and a BioRad ChemiDoc imaging system. Analysis was performed using ImageLab software.

### Quantification of MuSK in muscle lysate through ELISA

High binding 96-well half area plate (Greiner 675061) was coated with 1 μg/mL anti-MuSK Ig1 clone 3B5-hIgG4-S228P (argenx) overnight at 4°C. The plate was washed with wash buffer (0.05% Tween 20 in PBS). The plate was blocked in 1% casein in 1×PBS (Bio-Rad 1610783) for 1 h at RT, then washed. Samples were loaded onto the plate 7.5μg of protein per muscle lysate, 12-point standard curve of 10 ng/mL MuSK (Biotechne 9810-MK at 2-fold serial dilution) and incubated for 1 h at RT. The plate was washed and incubated with 1 μg/mL of biotinylated anti-MuSK IgG1 clone 3F6c-hIgG1-LALAdelK (argenx) for 1 h at 22°C. The plate was washed and incubated with 20 ng/mL streptavidin poly-HRP (Pierce 21140) for 30 min at 22°C. Following washing, total MuSK was detected with TMB substrate (Life Technologies N301). The reaction was stopped after 8 min with 0.5 M sulfuric acid and measured at OD450–620 nm with a plate reader.

### Quantification of 3B2 serum samples through ELISA

Quantification of 3B2 in serum samples was performed as described previously.^14^

### Statistics

All data was collated in Excel and analysis was performed using Graphpad prism V.9.4.1. Data was first tested for normal distribution (Shapir-Wilk test for normality) and then the appropriate test performed. Normally distributed data is presented as mean ± sd, while data that does not follow a normal distribution is presented as median and interquartile range. Operating scientists were unblinded during statistical analysis.

## Results

### Survival and bodyweight

**Figure 1:**
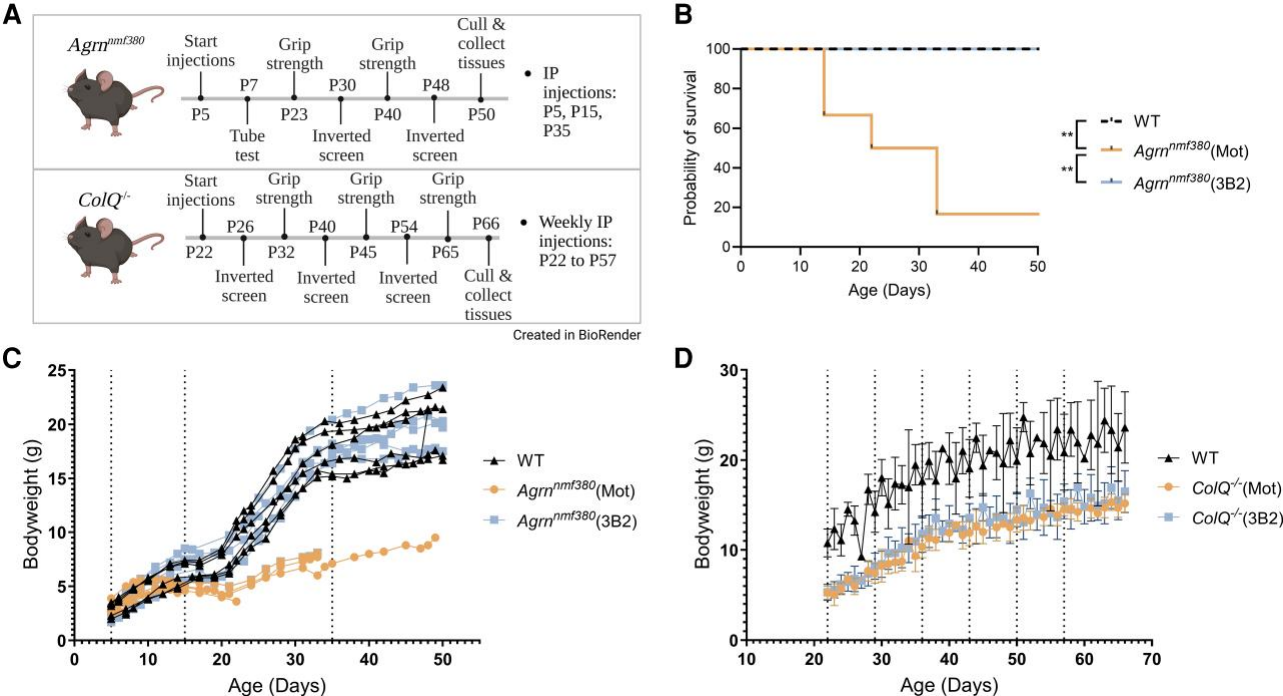
**Survival and bodyweight of *Agrn^nmf380^* and *ColQ^−/−^* mice.** (**A**) Treatment and testing protocol for *Agrn^nmf380^* and *ColQ*^−/−^ mice. Created in BioRender. Ho, K. (2026) https://BioRender.com/1vjypa9 (**B**) Kaplan-Meier curve showing **s**urvival of WT, *Agrn^nmf380^* (Mot), and *Agrn^nmf380^* (3B2) mice. Log-rank (Mantel-Cox) test. *x*^2^ of WT and *Agrn^nmf380^* (Mot) = 7.94, *x*^2^ of WT and *Agrn^nmf380^* (3B2) = 0.00, *x*^2^ of *Agrn^nmf380^* (Mot) and *Agrn^nmf380^* (3B2) = 7.94 (**C**) 3B2 treatment rescued bodyweights of *Agrn^nmf380^* mice. Each data point represents the bodyweight of each mouse. *F*(2, 278) = 55.60 (**D**) 3B2 treatment did not improve bodyweights of *ColQ^−/−^* mice. Each data point represents mean ± sd. *F*(2, 621) = 753.10 (**C**) and (**D**) Broken vertical lines indicate IP injections. 2-way mixed-effects analysis with Tukey’s multiple comparisons correction. (**B**) and (**C**) *n* = 6 mice per group. (**D**) WT *n* = 10, *ColQ*^−/−^ (Mot) *n* = 10, *ColQ*^−/−^ (3B2) *n* = 12. **P < 0.01.

Mice were assigned to one of three experimental conditions in our study. The first group was CMS mice treated with Fz-like domain MuSK agonist antibody (3B2). 3B2 is a human Immunoglobulin G1 (hIgG1) antibody with L234A and L235A (LALA) mutations, and deletion of the C-terminal lysine (delK). The second group was CMS mice treated with an isotype control antibody, Mota-hIgG1-LALA-delK (Mot). The final group was wild type (WT) litter mates used for healthy control comparisons. Mice assigned to 3B2 or Mot received an intraperitoneal (IP) injection on assigned days. The first IP injection was at a dosage of 20 mg/kg and all subsequent injections were 10 mg/kg (Supplementary Table 1). Animals underwent a series of behavioural assessments (hindlimb suspension, grip strength, and inverted screen tests) and blood draws. At the end of the study, mice were euthanized (CO_2_ followed by cervical dislocation), and tissues were weighed and collected. Due to the differing severities and presentations of the two animal models, slightly different protocols were used for each (Fig. 1A). We began treatment in the *ColQ^−/−^* mice at P22, after the phenotype had appeared and NMJs had matured, to determine whether the MuSK agonist antibody treatment could reverse disease symptoms for *COLQ-*CMS, making the experiment more clinically relevant. Since the *Agrn^nmf380^* mouse model has a severe phenotype, we began treatment early at P5.

The *AGRN-*CMS study was ended when mice were P50 (Fig. 1A). ELISAs for 3B2 confirmed the accumulation of the drug in the blood of the treated mice (Supplementary Table 2). Only one *Agrn^nmf380^* (Mot) mouse survived to endpoint, and half of the mice did not survive beyond P20. In contrast, all *Agrn^nmf380^* mice treated with 3B2, like WT mice, survived to the end of the study (Fig. 1B). *Agrn^nmf380^* mice had severely reduced bodyweights and 3B2 treatment rescued this to WT levels (Fig. 1C); we saw no sex differences in response to treatment (Supplementary Fig. 2).

In the *COLQ-*CMS study, all *ColQ^−/−^* and WT animals survived until the end of the study (P66) (Supplementary Fig. 1), and the drug was detectible in the serum at the end of the study (Supplementary Table 3). *ColQ^−/−^* mice also had reduced bodyweights and 3B2 treatment did not improve bodyweight (Fig. 1D); we saw no sex differences (Supplementary Fig. 3).

### Muscle strength

**Figure 2:**
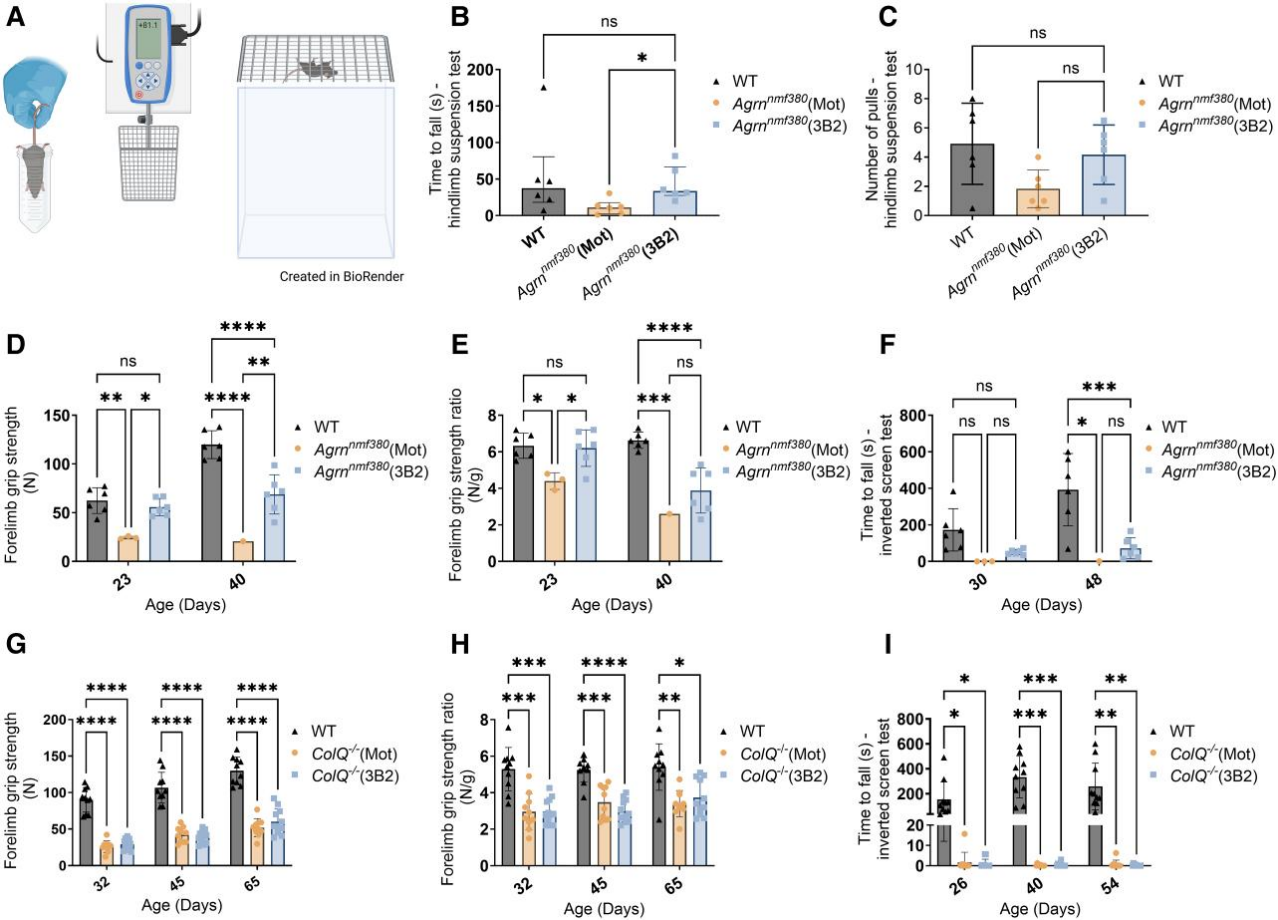
**Motor behavioural testing of *Agrn^nmf380^* and *ColQ^−/−^* mice.** (**A**) Schematic illustration of hindlimb suspension test, grip strength test and inverted screen test. Created in BioRender. Ho, K. (2026) https://BioRender.com/69cwunz (**B**) *Agrn^nmf380^* (3B2) mice held on for longer than *Agrn^nmf380^* (Mot) mice in the hindlimb suspension test at P7. Graph shows median ± IQR. Kruskal-Wallis test with Dunn’s multiple comparisons. *H*(2) = 7.06 (**C**) There were no significant differences in the number of pulls in the hindlimb suspension test. 1-way ANOVA with Tukey’s multiple comparisons correction. *F*(2,15) = 3.43 (**D**) 3B2 treatment improved forelimb grip strength of *Agrn^nmf380^* animals at P23 and P40. *F*(2, 12) = 25.77 (**E**) After normalization for bodyweight, 3B2 treatment improved grip strength of *Agrn^nmf380^* animals at P23. *F*(2,12) = 15.90 (**F**) Inverted screen test of WT, *Agrn^nmf380^* (Mot) and *Agrn^nmf380^* (3B2) mice at P30 and P48. *F*(2,22) = 14.55 (**G**) and (**H**) 3B2 treatment did not improve grip strength of *ColQ*^−/−^ mice at P32, P45 and P65. *F*(2,29) = 166.00, *F*(2,29) = 50.59 (**I**) 3B2 treatment did not improve ability to hold on in inverted screen test of *ColQ*^−/−^ mice at P26, P40 and P54. *F*(2,29) = 43.70 (**B**) to (**I**) Each data point represents each mouse. (**C**) to (**I**) Graphs show mean ± sd. (**D**) to (**I**) 2-way mixed-effects analysis with Tukey’s multiple comparisons correction. (**A**) and (**B**) *n* = 6 animals per group (**C**) to (**E**) At P23 & P30: WT *n* = 6, *Agrn^nmf380^* (Mot) *n* = 3, *Agrn^nmf380^* (3B2) *n* = 6. At P40 & P48: WT *n* = 6, *Agrn^nmf380^* (Mot) *n* = 1, *Agrn^nmf380^* (3B2) *n* = 6 (**F**) to (**H**) WT *n* = 10, *ColQ*^−/−^ (Mot) *n* = 10, *ColQ*^−/−^ (3B2) *n* = 12. *P < 0.05, **P < 0.005, ***P < 0.001, ****P < 0.0001, ns = non-significant.

*Agrn^nmf380^* mice were subjected to hindlimb suspension, grip strength, and inverted screen tests throughout the study (Fig. 2A). At P7, *Agrn^nmf380^* (3B2) mice held on with their hindlegs for longer than *Agrn^nmf380^* (Mot) mice (Fig. 2B), with no differences in number of pulls (Fig. 2C) or hindlimb suspension score (Supplementary Fig. 4) in the hindlimb suspension test. Grip strength and inverted screen tests for *Agrn^nmf380^* (Mot) mice were impacted by the poor survival of these mice, resulting in a low *n* for this condition. *Agrn^nmf380^* (3B2) mice had improved forelimb grip strength compared with *Agrn^nmf380^* (Mot) mice at P23 and P40 (Fig. 2D), although this improvement was not sustained following normalization to bodyweight at P40 (Fig. 2E). 3B2 treatment did not improve hindlimb grip strength in *Agrn^nmf380^* mice (Supplementary Fig. 5). 3B2 treatment did not improve time to fall (Fig. 2F) or holding impulse (Supplementary Fig. 5C) of *Agrn^nmf380^* mice in the inverted screen test. However, the performance between *Agrn^nmf380^* (Mot) and *Agrn^nmf380^* (3B2) mice was notable. At P30, *Agrn^nmf380^* (Mot) held on for an average of 0 s, while *Agrn^nmf380^* (3B2) mice held on for an average of 51.4 s. At P48, the one surviving *Agrn^nmf380^* (Mot) mouse held on for an average of 1.3 s, while *Agrn^nmf380^* (3B2) mice held on for an average of 72.3 s (Fig. 2F). A post-hoc analysis classifying mice as ‘non-holders’ (<2 s) and holders (>2 s) showed that 3B2 treatment may have improved inverted screen test performance in *Agrn^nmf380^* at P30 (Supplementary Table 4). It was not possible to examine this at P48 due to the low *n* number of *Agrn^nmf380^* (Mot) mice. We did not observe any sex differences of 3B2 treatment on *Agrn^nmf380^* muscle strength (Supplementary Fig. 6).

Unlike the *Agrn^nmf380^* mice, there was no improvement of forelimb grip strength in *ColQ^−/−^* mice animals with 3B2 treatment (Fig. 2G and H). Similar patterns were observed with hindlimb grip strength (Supplementary Fig. 5D and E). Likewise, 3B2 treatment did not improve inverted screen test performance in *ColQ^−/−^* mice (Fig. 2I and Supplementary Fig. 5F). We did not observe any sex differences of 3B2 treatment on *ColQ^−/−^* muscle strength (Supplementary Fig. 7).

### Muscle characteristics

**Figure 3:**
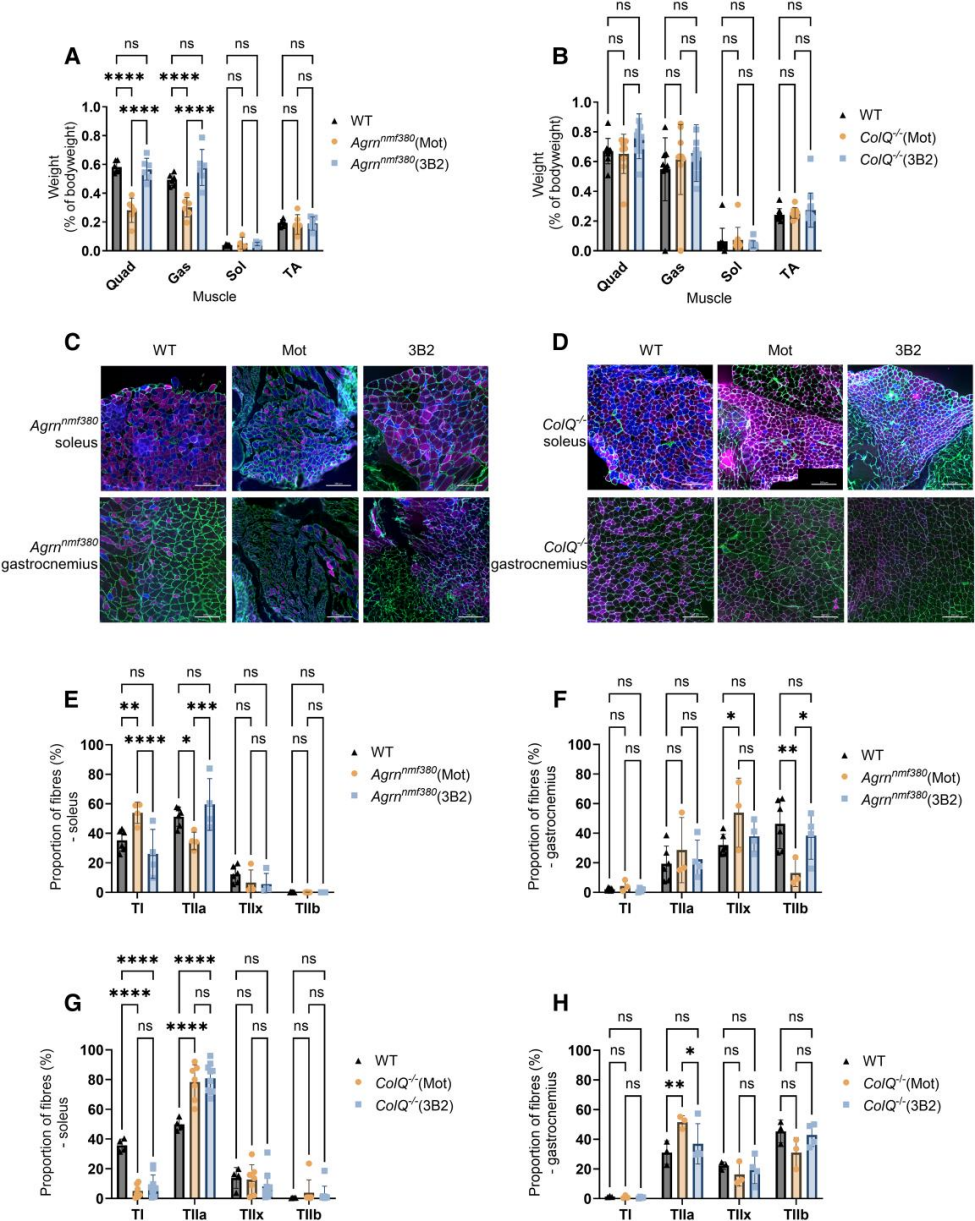
**Muscle characteristics of *Agrn^nmf380^* and *ColQ^−/−^* mice.** Quadriceps (Quad), gastrocnemius (Gas), soleus (Sol), and tibialis anterior (TA) muscles were weighed and normalized to bodyweight. (**A**) *Agrn^nmf380^* (3B2) mice had Quad and Gas muscle weights indistinguishable from WT. *F*(2,58) = 33.48 (**B**) There were no differences in muscle weight in the *ColQ-*CMS study. *F*(2120) = 2.03 (**C**) and (**D**) Cross sections of soleus and gastrocnemius muscles were stained to determine MHC type. Blue = type 1 (TI), magenta = type 2a (TIIa), green = type 2 × (TIIx & laminin), no labelling = type 2b (TIIb). Scale bar is 200 µm. (**E**) *Agrn^nmf380^* (3B2) mice demonstrated a full rescue of the fibre type switching observed in *Agrn^nmf380^* (Mot) mice in the soleus *F*(2,44) = 0.25 (**F**) and in the gastrocnemius. *F*(2,40) = 7.51e−021 (**G**) 3B2 treatment did not reverse the slow to fast MHC fibre type switching observed in *ColQ^−/−^* (Mot) mice. *F*(2,72) = 5.22E-014 (**H**) *ColQ*^−/−^ (3B2) mice demonstrated a full rescue of the fibre type switching observed in the *ColQ*^−/−^ (Mot) mice in the gastrocnemius. *F*(2,28) = 8.21e−014 (**B**) to (**D**) and (**E**) to (**G**) Each data point represents each mouse. Graphs show mean ± sd. 2-Way ANOVA with Tukey’s multiple comparisons correction. (**A**) *n* = 6 animals per group (**B**) WT *n* = 10, *ColQ*^−/−^ (Mot) *n* = 10, *ColQ*^−/−^ (3B2) *n* = 12 (**E**) WT *n* = 6, *Agrn^nmf380^* (Mot) *n* = 4, *Agrn^nmf380^* (3B2) *n* = 4 (**F**) WT *n* = 6, *Agrn^nmf380^* (Mot) *n* = 4, *Agrn^nmf380^* (3B2) *n* = 4 (**G**) WT *n* = 4, *ColQ*^−/−^ (Mot) *n* = 7, *ColQ*^−/−^ (3B2) *n* = 10 (**H**) WT *n* = 3, *ColQ*^−/−^ (Mot) *n* = 3, *ColQ*^−/−^ (3B2) *n* = 4 *P < 0.05, **P < 0.005, ***P < 0.001, ****P < 0.0001, ns = non-significant.

At the end of the experiments, muscle tissue was collected and weighed (Supplementary Fig. 8A and B). When calculated relative to bodyweight, 3B2 treatment rescued muscle weight to WT levels (Fig. 3A). Both WT and *Agrn^nmf380^* (3B2) animals had significantly heavier quadriceps (Quad) and gastrocnemius (Gas) muscles than *Agrn^nmf380^* (Mot) animals (Fig. 3A). The difference in muscle weight normalized to bodyweight suggested that the increased muscle weight cannot be fully explained by variations in age or overall bodyweight of the animals. Following normalization for bodyweight, 3B2-treated *ColQ^−/−^* mice had a partial rescue in quad muscle weight compared with *ColQ^−/−^* (Mot) mice (Fig. 3B). No sex differences in muscle weight were detected (Supplementary Figs. 2 and 3).

We examined myosin heavy chain (MHC) type expression in the soleus and gastrocnemius muscles of *Agrn^nmf380^* and *ColQ^−/−^* mice (Fig. 3C and D). *Agrn^nmf380^* mice exhibited a fast to slow fibre type shift in the soleus (Fig. 3E), as previously described in this model and in patients.^15,30-32^ When we examine MHC type expression in the gastrocnemius, *Agrn^nmf380^* mice had a similar fast to slower fibre type shift, as seen with a shift from type 2b (TIIb) to type 2 × (TIIx) fibres (Fig. 3F). This abnormal fibre type shift in the soleus and gastrocnemius was reversed in *Agrn^nmf380^* mice treated with 3B2 (Fig. 3E and F).

In the soleus, *Agrn^nmf380^* (Mot) mice had smaller muscle fibres compared with WT, while *Agrn^nmf380^* (3B2) mice had no difference compared with WT (Table 1). When broken down into fibre types, *Agrn^nmf380^* (Mot) muscle had smaller type 2a (TIIa), TIIx and TIIb fibres compared with WT, while *Agrn^nmf380^* (3B2) muscle had no difference in TIIx and TIIb fibres (Table 1). In the gastrocnemius, *Agrn^nmf380^* (Mot) mice had smaller muscle fibres and smaller TI, TIIa, TIIx and TIIb fibres. 3B2 treatment rescued the size of all muscle fibres (Table 1). This improvement in fibre size with 3B2 treatment was also seen in the quadriceps muscle of *Agrn^nmf380^* mice with H & E histochemistry (Supplementary Fig. 9A and B).

**Table 1:** Fibre feret diameter by MHC type

		WT	Mot	3B2
*Agrnnmf380* soleus	total	19.9 ± 4.5	11.9 ± 4.0**	18.6 ± 7.0
	TI	21.2 ± 4.7	15.2 ± 4.9	22.9 ± 5.0
	TIIa	19.9 ± 3.9	12.4 ± 4.1***	16.2 ± 5.2*
	TIIx	17.8 ± 5.0	10.8 ± 3.3**	16.8 ± 7.5
	TIIb	17.1 ± 4.5	11.0 ± 2.9*	18.6 ± 7.1
*Agrnnmf380* gastroc	total	28.7 ± 8.6	15.9 ± 5.4***	27.0 ± 7.9^###^
	TI	25.1 ± 4.6	14.0 ± 4.2**	23.6 ± 11.2^##^
	TIIa	23.2 ± 5.5	13.2 ± 3.6**	25.0 ± 8.8^###^
	TIIx	29.1 ± 6.6	16.7 ± 5.6***	26.5 ± 7.8^###^
	TIIb	31.3 ± 9.9	17.5 ± 5.6***	30.4 ± 4.9^###^
*ColQ−/−* soleus	total	28.2 ± 4.9	24.9 ± 6.4**	26.9 ± 6.6
	TI	26.0 ± 6.1	18.3 ± 4.8***	16.1 ± 5.4**
	TIIa	26.2 ± 3.5	21.4 ± 3.3**	24.9 ± 5.5
	TIIx	27.7 ± 7.0	23.5 ± 3.5**	25.3 ± 5.3
	TIIb	20.6 ± 9.0	26.1 ± 5.0	25.9 ± 6.3
*ColQ−/−* gastroc	total	30.3 ± 9.3	27.8 ± 6.4	25.7 ± 5.7
	TI	26.5 ± 6.6	19.9 ± 7.8	17.3 ± 4.7
	TIIa	26.1 ± 7.5	26.9 ± 6.3	23.8 ± 5.2
	TIIx	35.1 ± 9.6	31.2 ± 9.7	28.5 ± 10.2*
	TIIb	34.3 ± 9.4	30.3 ± 6.9	32.0 ± 7.2

Fibres were traced using laminin as a guide, and the area relative to the MHC type was determined. Data shown as mean **±** sd. WT *n* = 6, *Agrn^nmf380^* (Mot) *n* = 3 (note unequal sexes), *Agrn^nmf380^* (3B2) *n* = 4, *ColQ^−/−^* WT *n* = 4, *ColQ*^−/−^ (Mot) *n* = 7, 4 *ColQ*^−/−^ (3B2) *n* = 10. *Significantly different from WT, ^#^significantly different from Mot. *P < 0.05, 5 **/##*P* < 0.005, ***/###*P* < 0.001.

As previously shown^20,24^, *ColQ*^−/−^ (Mot) mice had a slow to fast fibre type shift, with a lower proportion of TI fibres and higher proportion of TIIa fibres in the soleus (Fig. 3G), and a higher proportion of TIIa fibres in the gastrocnemius when compared with WT (Fig. 3H). This abnormal fibre type shift was not reversed with 3B2 treatment in the soleus (Fig. 3F), but was reversed in the gastrocnemius (Fig. 3G). Similar to *Agrn^nmf380^* (Mot) mice, *ColQ^−/−^* (Mot) mice had smaller muscle fibres in the soleus compared with WT, while *ColQ^−/−^* (3B2) mice had no difference compared with WT (Table 1). When broken down into fibre types, *ColQ^−/−^* (Mot) soleus had smaller TI, TIIa and TIIx fibres, while *ColQ^−/−^* (3B2) soleus had no difference in TIIa and TIIx fibres (Table 1). There were no differences in gastrocnemius fibre feret between WT, *ColQ^−/−^* (Mot) and *ColQ^−/−^* (3B2) mice (Table 1).

### NMJ structure

**Figure 4:**
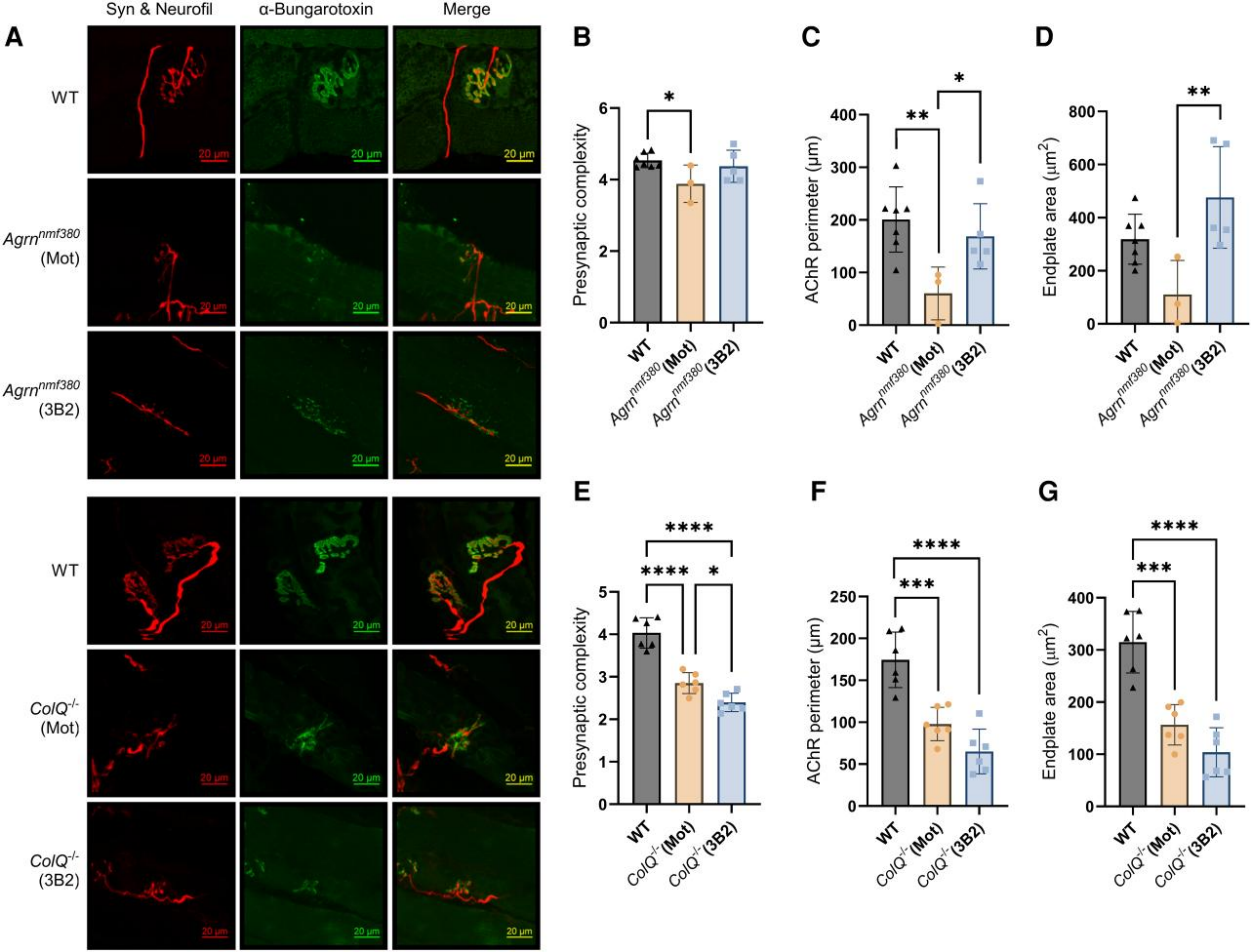
**NMJ morphological analysis of *Agrn^nmf380^* and *ColQ^−/−^* mice.** (**A**) NMJs in whole soleus muscles were labelled using α-Bungarotoxin, synaptophysin (Syn), and neurofilament (Neurofil). Maximum intensity projection images were analysed to calculate various morphological features of the NMJs. Scale bar is 20 µm. (**B**) No differences were found between WT and *Agrn^nmf380^* (3B2) in presynaptic complexity. *F*(2,12) = 4.40 (**C**) 3B2 treatment improved AChR perimeter. *F*(2,12) = 7.04 and (**D**) endplate area in *Agrn^nmf380^* mice. *F*(2,12) = 7.85 (**E**) 3B2 treatment reduced presynaptic complexity *F*(2,15) = 53.47, (**F**) and had no effect on AChR perimeter *F*(2,15) = 25.91 and (**G**) endplate area in *ColQ^−/−^* mice. *F*(2,15) = 30.40 (**B**) to (**G**) Each data point represents each mouse. Graphs show mean ± sd. Nested 1-way ANOVA with Tukey’s multiple comparisons correction. (**B**) to (**D**) WT *n* = 7, *Agrn^nmf380^* (Mot) *n* = 3, *Agrn^nmf380^* (3B2) *n* = 5. An average of 9.93 NMJs were analysed per mouse. (**E**) to (**G**) *n* = 6 animals per group. An average of 20.72 NMJs were analysed per mouse. *P < 0.05, **P < 0.005, ***P < 0.001, ****P < 0.0001.

To determine changes in NMJ morphology in the soleus muscle, analysis was performed on maximum intensity projections of NMJs taken using confocal microscopy (Fig. 4A) and analysed as previously described^17,29^ (Supplementary Tables 5 and 6). Overall, the three *Agrn^nmf380^* (Mot) mice that survived until the end of the study had abnormal NMJ morphology in the postsynaptic variables. Presynaptic complexity, calculated as reported previously,^17,29^ showed differences (Fig. 4B), while many other presynaptic variables showed trends towards abnormal NMJ morphology (Supplemental Table 5). 3B2 treatment improved numerous postsynaptic measures, including AChR perimeter (Fig. 4C) and endplate area (Fig. 4D). *ColQ^−/−^* mice had abnormal NMJ morphology in almost all variables, which were not improved by 3B2 treatment, in contrast to *Agrn^nmf38^* mice (Fig. 4E to G, Supplementary Table 6).

### Quantification of MuSK and phosphorylated MuSK

**Figure 5:**
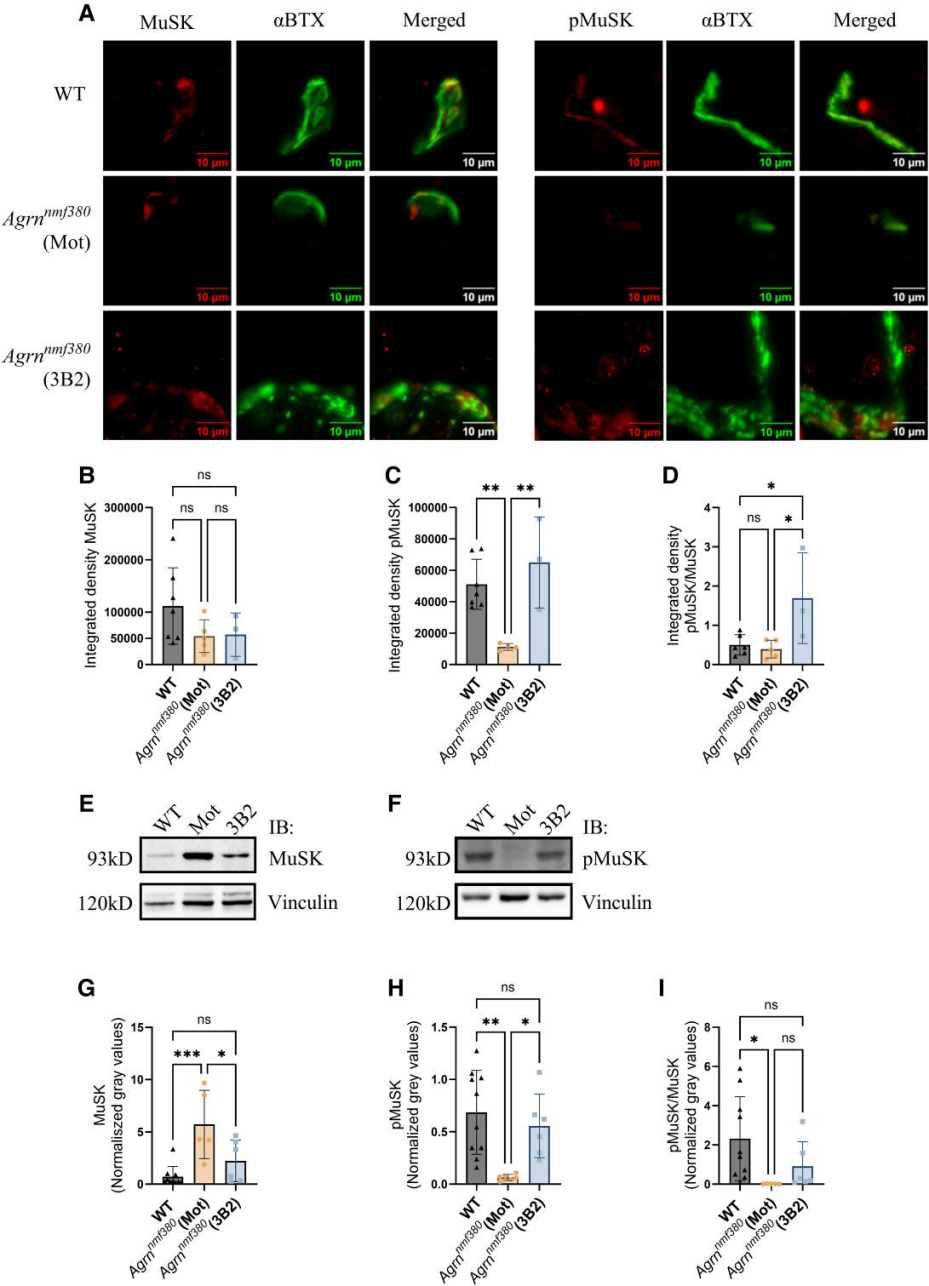
**Synaptic MuSK and pMuSK in *Agrn^nmf380^* mice.** (**A**) Gastrocnemius muscles were labelled with α-bungarotoxin (α-BTX), MuSK, and pMuSK, and the integrated density determined. (**B**) No differences in MuSK at the NMJ were detected between groups. *F*(2,12) = 1.81 (**C**) 3B2 treatment increased pMuSK *F*(2,11) = 10.14 (**D**) and pMuSK/MuSK at the NMJ in *Agrn^nmf380^* mice. *F*(2,11) = 6.22 (**E**) Representative MuSK and (**F**) pMuSK Western immunoblot (IB) of TA lysates from WT, *Agrn^nmf380^* (Mot) and *Agrn^nmf380^* (3B2) mice. (**G**) MuSK levels were increased in *Agrn^nmf380^* (Mot) mice, which was reversed with 3B2 treatment. *F*(2,18) = 10.56 (**H**) pMuSK levels were reduced in *Agrn^nmf380^* (Mot) mice, which was reversed with 3B2 treatment. *F*(2,19) = 7.4 (**I**) pMuSK/MuSK levels were redueced in *Agrn^nmf380^* (Mot) mice. *F*(2,19) = 7.44 (**B**) to (**D**) and (**G**) to (**I**) Each data point represents each mouse. Graphs show mean ± sd. 1-way ANOVA with Tukey’s multiple comparisons correction. (**B**) to (**D**) WT *n* = 7, *Agrn^nmf380^* (Mot) *n* = 5, *Agrn^nmf380^* (3B2) *n* = 3 (**G**) and (**I**) WT *n* = 10, *Agrn^nmf380^* (Mot) *n* = 5 *Agrn^nmf380^* (3B2) *n* = 6 (**H**) WT *n* = 10, *Agrn^nmf380^* (Mot) *n* = 7, *Agrn^nmf380^* (3B2) *n* = 7. *P < 0.05, **P < 0.005, ***P < 0.001, ns = non-significant. See Supplementary Figs 10 and 11 for uncropped blots.

MuSK agonist antibodies that bind the Fz-like domain of MuSK stimulate MuSK dimerization and phosphorylation *in vivo.*^13^ We investigated the effects of 3B2 treatment on MuSK and pMuSK in two ways. Firstly, we performed immunofluorescence labelling at the NMJ in the gastrocnemius muscle (Fig. 5A). In *Agrn^nmf380^* animals, there was no reduction in MuSK expression at the NMJ, nor a change with 3B2 treatment (Fig. 5B). There was, however, a reduction in pMuSK levels in *Agrn^nmf380^* mice that was rescued by 3B2 treatment (Fig. 5C). When the ratio of pMuSK/MuSK was determined, there was a 4.3-fold increase in *Agrn^nmf380^* (3B2) mice (Fig. 5D). We also examined MuSK and pMuSK levels in TA muscle lysates using Western blot (Fig. 5E to I, Supplementary Figs. 10 and 11). *Agrn^nmf380^* (Mot) mice had an 8.0-fold increase in MuSK (Fig. 5G) and a 0.1-fold decrease in pMuSK compared with WT (Fig. 5H), which were both rescued with 3B2 treatment. When we normalized pMuSK to MuSK levels, *Agrn^nmf380^* (Mot) mice had a 0.007-fold decrease in pMuSK/MuSK levels compared with WT, while *Agrn^nmf380^* (3B2) mice had no significant differences in pMuSK/MuSK levels compared with WT (Fig. 5I). We also quantified MuSK levels in the TA with an enzyme-linked immunosorbent assay (ELISA) and found an increase in MuSK in *Agrn^nmf380^* (Mot) mice, which was normalized with 3B2 treatment (Supplementary Fig. 14A), consistent with our Western blot results.

**Figure 6:**
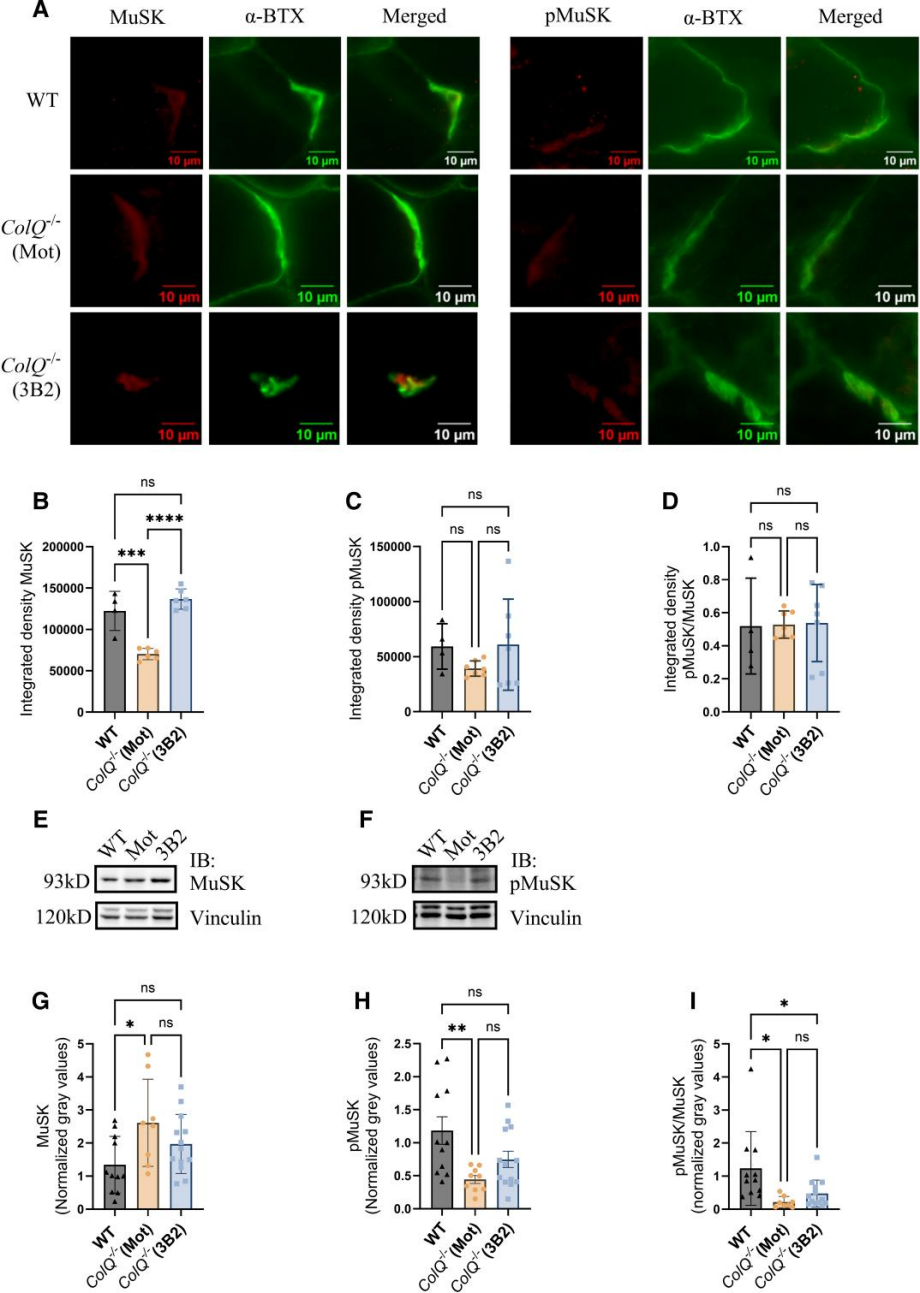
**MuSK and pMuSK in *ColQ*^−/−^ mice.** (**A**) Gastrocnemius muscles were labelled with α-bungarotoxin (α-BTX), MuSK, and pMuSK, and the integrated density determined. (**B**) 3B2 treatment rescued total MuSK levels at the NMJ in *ColQ*^−/−^ mice. *F*(2,13) = 34.84 (**C**) 3B2 treatment did not increase pMuSK *F*(2,15) = 1.21 or (**D**) pMuSK/MuSK ratio at the NMJ in *ColQ*^−/−^ mice. *F*(2,15) = 0.01 (**E**) Representative MuSK and (**F**) pMuSK Western immunoblot (IB) of TA lysates from WT, *ColQ*^−/−^ (Mot) and *ColQ*^−/−^ (3B2) mice. (**G**) *ColQ*^−/−^ (Mot) mice had higher levels of MuSK compared with WT. *F*(2,29) = 3.75 (**H**) 3B2 treatment did not increase pMuSK *F*(2,30) = 5.67 or (**I**) pMuSK/MuSK levels in *ColQ*^−/−^ mice. *F*(2,29) = 5.60 (**B**) to (**D**) and (**G**) to (**I**) Each data point represents each mouse. Graphs show mean ± sd. 1-way ANOVA with Tukey’s multiple comparisons correction. (**B**) to (**D**) WT *n* = 4, *ColQ*^−/−^ (Mot) *n* = 6, *ColQ*^−/−^ (3B2) *n* = 6. (**G**) and (**I**) WT *n* = 11, *ColQ*^−/−^ (Mot) *n* = 8, *ColQ*^−/−^ (3B2) *n* = 13 (**H**) WT *n* = 11, *ColQ*^−/−^ (Mot) *n* = 9, *ColQ*^−/−^ (3B2) *n* = 13. *P < 0.05, **P < 0.005, ***P < 0.001, ****P < 0.0001, ns = non-significant. See Supplementary Figs 12 and 13 for uncropped blots.

In *ColQ^−/−^* mice, we likewise performed immunofluorescent labelling of MuSK and pMuSK at the NMJ in the gastrocnemius muscle (Fig. 6A). *ColQ^−/−^* mice had a 0.6-fold decrease in MuSK compared with WT mice, which was rescued to WT levels with 3B2 treatment (Fig. 6B). We further quantified extrasynaptic MuSK through immunofluorescent labelling and found no differences between experimental groups (Supplementary Fig. 14B). There was no reduction in pMuSK (Fig. 6C) or the pMuSK/MuSK ratio (Fig. 6D) in *ColQ*^−/−^ mice. Western Blot of the TA lysate (Fig. 6E and F, Supplementary Figs. 12 and 13) showed that *ColQ*^−/−^ (Mot) mice had a 1.9-fold increase in MuSK levels and 0.4-fold decrease in pMuSK levels than WT (Fig. 6F), with no improvement with 3B2 treatment. ELISA quantification of MuSK showed that there was an increase in total MuSK in *ColQ*^−/−^ (3B2) mice when compared with both WT and *ColQ*^−/−^ (Mot) mice (Supplementary Fig. 14C).

## Discussion

CMS are a group of genetic disorders in which mutations in genes encoding proteins at the NMJ impair neuromuscular transmission. *AGRN-*CMS and *COLQ-*CMS patients have abnormal NMJ morphology and function, presenting with fatigable muscle weakness. The most common CMS treatment, AChE inhibitors, are ineffective and sometimes detrimental for both *AGRN-* and *COLQ-*CMS.^33^ MuSK agonist antibodies that target the Fz-like domain and stimulate MuSK phosphorylation have improved survival, weight gain, and motor behaviour in *Dok7*-CMS mice.^13,14^ In some cases, these treated mice have proceeded to become fertile adults.^13,14^ We noted many similarities in the ARGX-119 treatment of *Dok7-*CMS mice with our 3B2 treatment of *Agrn-*CMS mice. Perhaps the most exciting, both *Dok7-* and *Agrn-*CMS survival was fully rescued with MuSK Fz-like domain agonist antibody treatment. Although our *n* number for the *Agrn^nmf380^* (Mot) was low due to reduced survival, the 3 mice that did survive allowed us to make numerous important comparisons between Mot- and 3B2-treated *Agrn-*CMS mice, such as muscle fibre typing and NMJ morphology. Since this was not possible to do in the *Dok7-*CMS study, our study provides further valuable insight into MuSK Fz-like domain agonist antibody as a CMS treatment.

This is the first time an agonist antibody treatment has been shown to rescue the phenotype in *Agrn-*CMS mice. Coupled with an easy delivery system, we hypothesize that 3B2 may be a therapeutic option for patients with *AGRN*-CMS. *Agrn^nmf380^* mice have a partial loss of function of agrin, resulting in reduced survival, bodyweight, and motor strength when assessed through grip strength and inverted screen tests. We and others have reported that untreated *Agrn*^nmf380^ mice often die from P12 and onwards.^15,17^ We report remarkable rescues in survival and bodyweight in our *Agrn^nmf380^* mice with Fz-like MuSK agonist antibody treatment. 3B2 treatment increased time to fall in the hindlimb suspension test and forelimb grip strength in *Agrn^nmf380^* mice. However, they did not experience a full rescue of forelimb grip strength back to WT levels at the later time point of P40. This may be indicative that more frequent dosing of 3B2 is required in the *Agrn*^nmf380^ mice to maintain WT level forelimb grip strength. In the inverted screen test, 3B2 treatment did not significantly improve latency to fall or holding impulse. However, the differences we saw in *Agrn^nmf380^* mice with 3B2 treatment (0 s to 51.4 s) were an extremely biologically relevant improvement. When a post-hoc analysis was performed by classifying mice as ‘non-holders’ (<2 s) and holders (>2 s), 3B2 treatment significantly increased the number of *Agrn^nmf380^* mice classified as holders at P30. Future studies may benefit by analyzing this test in groups of non-holders, holders, and extended holders, to avoid missing what may be relevant results. In addition, beginning treatment even earlier could result in more beneficial effects.

An increase in muscle weight of *Agrn^nmf380^* (3B2) mice back to WT levels is not surprising given their increase in bodyweight; this improvement remained after normalizing to bodyweight, suggesting that strength gains are also due to an improvement intrinsic to the nerve-muscle complex.

As reported previously,^17^  *Agrn^nmf380^* mice had smaller muscle fibres in the soleus, gastrocnemius and quadriceps, and in the soleus, had smaller TIIa, TIIx, and TIIb fibres, while TI fibres remained unchanged. These findings are in line with the fast to slow fibre type shift we observed in these animals. In the gastrocnemius, all muscle fibre types were smaller in the *Agrn^nmf380^* mice. The differences between *Agrn^nmf380^* (Mot) and WT mice were almost completely reversed in the *Agrn^nmf380^* (3B2) mice. While we have previously observed rescues of these features in *Agrn^nmf380^* animals with treatment of a modified form of agrin,^17^ this is the first time we have seen a recovery back to WT levels. The MHC type is influenced by several factors, including neural input, in the case of NMJ dysfunction this is perturbed, and fibre-type conversion or a shift to an undifferentiated state can be observed. The rescue of this with 3B2 suggests an improvement in the neural input secondary to NMJ improvement. The mosaicism of MHC types in a muscle is important to preserve energy efficiency, adaptability, precision and control of movement.^34^

This was the first time MuSK agonist antibody treatment has been shown to improve NMJ morphology in a CMS mouse model. In the case of *Agrn^nmf380^* (3B2) mice, we observed morphology improvements in postsynaptic components of the NMJ. This is in line with 3B2’s proposed mechanism of action. Similarly, MuSK agonist antibody treatment has been reported to improve NMJ morphology in mouse models of other neuromuscular diseases, including myasthenia gravis,^35^ amyotrophic lateral sclerosis^36-38^ and spinal muscular atrophy.^39^ Both Western blot and immunofluorescent labelling showed *Agrn^nmf380^* (Mot) mice had severely reduced pMuSK levels, which were rescued with 3B2 treatment. Conversely, Western blot and ELISA showed *Agrn*^nmf380^ (Mot) mice had higher total MuSK levels than both *Agrn^nmf380^* (3B2) and WT animals. MuSK expression is dramatically induced in adult muscle following denervation,^40^ which has been previously reported in *Agrn^nmf380^* mice^15^ and in *AGRN-*CMS patients.^41^ Alternatively, these high levels could be due to the young age at which *Agrn^nmf380^* (Mot) mice died. During the first weeks of postnatal development, the NMJ undergoes intensive maturation, and MuSK is highly involved in the prepatterning of muscles, while in adult mice, expression is limited to the subsynaptic nuclei in the central region of the muscle.^8,42^ MuSK expression has been shown to dramatically downregulate shortly after birth in rats.^40^

While ColQ does not sit in the agrin/LRP4/MuSK pathway, it has been reported to bind to the Ig-like 1 and Fz-like domains of MuSK, stabilizing it,^25^ and its absence results in decreased muscle membrane-bound MuSK.^19,20,25^ However, this control of MuSK by ColQ has also been hypothesized to act via LRP4.^43^ In addition, the binding of ColQ to MuSK inhibits agrin signalling,^25^ demonstrating the opposing ways ColQ maintains NMJ homeostasis. This integral relationship between ColQ and MuSK led us to hypothesize that 3B2 treatment might stabilize and phosphorylate MuSK, thus providing a therapeutic benefit to *ColQ*^−/−^ mice.

As previously reported, *ColQ*^−/−^ mice had decreased bodyweight and motor strength, a slow to fast fibre type switch in the soleus and gastrocnemius, and abnormal NMJ morphology.^20,24^ We found that while 3B2 treatment reversed the abnormal fibre type switch in the gastrocnemius and increased MuSK levels, it was unable to rescue most phenotypic and cellular characteristics, including bodyweight, grip strength, muscle fibre size, pMuSK levels and NMJ morphology. Thus, this treatment may not be appropriate for *COLQ*-CMS. Because the absence of ColQ leads to reduced synaptic localization of MuSK,^19,24^  *ColQ*^−/−^ mice had lower pMuSK levels in the TA lysate. However, without MuSK stabilization in the absence of ColQ, 3B2 treatment may not be able to increase pMuSK levels to provide therapeutic benefit in *ColQ*^−/−^ mice. While our Western blots showed higher levels of MuSK in *ColQ*^−/−^ (Mot) mice, ELISA and immunostaining showed that 3B2 treatment increased total MuSK levels, but this did not improve the phenotypes of *ColQ*^−/−^ mice. These differences may be due to different MuSK antibodies being used in the two assays. We investigated whether the changes in MuSK levels in *ColQ*^−/−^ mice were due to the increase of extrasynaptic MuSK levels, but this was not the case.

In addition, 3B2 treatment did not rescue any of the NMJ morphological changes in the *ColQ*^−/−^ (Mot) mice. While the absence of ColQ has been shown to reduce pMuSK levels *in vitro*,^43^ this is the first time it has been reported *in vivo.* However, at both the synapse and in the muscle overall, 3B2 increased MuSK levels, which was not accompanied by an increase in pMuSK.

The main mechanism of disease in *COLQ-*CMS is the absence of AChE, leading to an excess of ACh in the synaptic cleft, repetitive action potentials at the muscle fibre, and degeneration of the postjunctional folds. The stabilization of MuSK on the muscle membrane by ColQ may explain some of the common clinical features between *COLQ-*CMS and CMS subtypes directly impacted by the agrin/LRP4/MuSK pathway, such as *AGRN-* and *DOK7-*CMS.^44-47^ Furthermore, the most beneficial treatment for *COLQ-*CMS and CMS subtypes directly impacted by the AGRIN/LRP4/MuSK pathway is salbutamol, a treatment that helps to increase AChR clusters,^24,48,49^ although its mechanism of action is unknown.

Unfortunately, 3B2 treatment did not improve most of the cellular and phenotypic characteristics in *ColQ^−/−^* mice, likely due to the reduction in pMuSK not being the main mechanism of disease of *COLQ-*CMS. These divergent results in *Agrn^nmf380^* and *ColQ*^−/−^ mice are supported by the well-known fact that treatments for CMS are specific to the mutated gene, and administering the wrong treatment to a patient can be detrimental. Thus, it remains important that new CMS treatments be tested in a variety of CMS models before being given to patients. In this case, we would hypothesize that 3B2 may not be beneficial for *COLQ-*CMS patients but may be extremely beneficial to *AGRN*-CMS patients. It will be important to test this compound in animal models with other mutations: we would suggest other components that act in the agrin/LRP4/MuSK pathway as a good place to start. Indeed, clinical trials with a similar MuSK agonist antibody^14^ are being conducted for patients with *DOK7-*CMS (NCT06436742). Studies also suggest that MuSK agonist antibodies may have use beyond CMS, including MuSK-myasthenia gravis, amyotrophic lateral sclerosis, and spinal muscle atrophy.^35-39,50^

In this study, we tested the hypothesis that 3B2, a MuSK agonist antibody and derivative of ARGX-119, would be beneficial in animal models of CMS with defects in proteins that directly and indirectly interact with the agrin/LRP4/MuSK pathway. While the different results in *Agrn^nmf380^* and *ColQ*^−/−^ mice likely stem from the distinct functions of agrin and ColQ, the two models received different treatment protocols due to phenotype severity. *Agrn^nmf380^* mice began treatment before the phenotype was visually evident at P5, while treatment of the *ColQ*^−/−^ mice began at P22, after symptoms, notably their smaller size, was evident, and after NMJs in WT mice appear mature. Therefore, we cannot exclude the possibility that earlier dosing of the *ColQ*^−/−^ mice may lead to improved outcomes. However, we previously rescued some CMS features in *ColQ*^−/−^ mice using salbutamol, initiating treatment at a similar age,^24^ demonstrating that NMJs remain tractable post-symptom onset. Another difference between the treatment protocols was the dosing regimen: *Agrn^nmf380^* mice were injected at P5, P15 and P35, whereas *ColQ*^−/−^ mice were injected weekly. While we think these differences do not impact the overall conclusions of this study, follow-up experiments should be performed to determine whether alternative dosing might improve the deficits of *ColQ*^−/−^ mice, and whether starting treatment later would still rescue *Agrn^nmf380^* mice. In addition, future studies should seek to examine if response to 3B2 differs between muscles.

## Conclusions

In conclusion, successful treatment of CMS-mice with a MuSK agonist antibody (3B2) is mutation-dependent. In *Agrn^nmf380^* mice, treatment with the MuSK agonist antibody rescued NMJ morphology, cellular disease, and life span, while similar benefits were not found in *ColQ*^−/−^ mice. These results highlight the importance of identifying the genetic cause of CMS to effectively treat patients.

## Supplementary Material

fcag115_Supplementary_Data

## Data Availability

The raw data supporting the conclusions of this article is available from the corresponding author, upon reasonable request.

## References

[1] Slater CR . Reliability of neuromuscular transmission and how it is maintained. Handb Clin Neurol. 2008;91:27–101. 18631840 10.1016/S0072-9752(07)01502-3

[2] Ohno K, Ohkawara B, Shen XM, Selcen D, Engel AG. Clinical and pathologic features of congenital myasthenic syndromes caused by 35 genes-A comprehensive review. Int J Mol Sci. 2023;24(4):3730. 36835142 10.3390/ijms24043730 PMC9961056

[3] Sander A, Hesser BA, Witzemann V. MuSK induces in vivo acetylcholine receptor clusters in a ligand-independent manner. J Cell Biol. 2001;155(7):1287–1296. 11748247 10.1083/jcb.200105034 PMC2199313

[4] Zong Y, Jin R. Structural mechanisms of the agrin-LRP4-MuSK signaling pathway in neuromuscular junction differentiation. Cell Mol Life Sci. 2013;70(17):3077–3088. 23178848 10.1007/s00018-012-1209-9 PMC4627850

[5] Bergamin E, Hallock PT, Burden SJ, Hubbard SR. The cytoplasmic adaptor protein Dok7 activates the receptor tyrosine kinase MuSK via dimerization. Mol Cell. 2010;39(1):100–109. 20603078 10.1016/j.molcel.2010.06.007 PMC2917201

[6] Hallock PT, Xu CF, Park TJ, Neubert TA, Curran T, Burden SJ. Dok-7 regulates neuromuscular synapse formation by recruiting Crk and Crk-L. Genes Dev. 2010;24(21):2451–2461. 21041412 10.1101/gad.1977710 PMC2964755

[7] Borges LS, Yechikhov S, Lee YI, et al Identification of a motif in the acetylcholine receptor beta subunit whose phosphorylation regulates rapsyn association and postsynaptic receptor localization. J Neurosci. 2008;28(45):11468–11476. 18987183 10.1523/JNEUROSCI.2508-08.2008 PMC2606670

[8] Burden SJ, Yumoto N, Zhang W. The role of MuSK in synapse formation and neuromuscular disease. Cold Spring Harb Perspect Biol. 2013;5(5):a009167. 23637281 10.1101/cshperspect.a009167 PMC3632064

[9] Friese MB, Blagden CS, Burden SJ. Synaptic differentiation is defective in mice lacking acetylcholine receptor beta-subunit tyrosine phosphorylation. Development. 2007;134(23):4167–4176. 17959719 10.1242/dev.010702

[10] Ohno K, Ito M, Ohkawara B. Review of 40 genes causing congenital myasthenic syndromes. J Hum Genet. 2025;70(6):433-447. 10.1038/s10038-025-01355-9 40533459

[11] Beeson D, Higuchi O, Palace J, et al Dok-7 mutations underlie a neuromuscular junction synaptopathy. Science. 2006;313(5795):1975–1978. 16917026 10.1126/science.1130837

[12] Müller JS, Herczegfalvi A, Vilchez JJ, et al Phenotypical spectrum of DOK7 mutations in congenital myasthenic syndromes. Brain. 2007;130(Pt 6):1497–1506. 17439981 10.1093/brain/awm068

[13] Oury J, Zhang W, Leloup N, et al Mechanism of disease and therapeutic rescue of Dok7 congenital myasthenia. Nature. 2021;595(7867):404–408. 34163073 10.1038/s41586-021-03672-3 PMC8277574

[14] Vanhauwaert R, Oury J, Vankerckhoven B, et al ARGX-119 is an agonist antibody for human MuSK that reverses disease relapse in a mouse model of congenital myasthenic syndrome. Sci Transl Med. 2024;16(765):eado7189. 10.1126/scitranslmed.ado7189 39292800

[15] Bogdanik LP, Burgess RW. A valid mouse model of AGRIN-associated congenital myasthenic syndrome. Hum Mol Genet. 2011;20(23):4617–4633. 21890498 10.1093/hmg/ddr396 PMC3209832

[16] Xi J, Yan C, Liu WW, et al Novel SEA and LG2 Agrin mutations causing congenital Myasthenic syndrome. Orphanet J Rare Dis. 2017;12(1):182. 29258548 10.1186/s13023-017-0732-z PMC5735900

[17] Spendiff S, Howarth R, McMacken G, et al Modulation of the acetylcholine receptor clustering pathway improves neuromuscular junction structure and muscle strength in a mouse model of congenital myasthenic syndrome. Front Mol Neurosci. 2020;13:594220. 33390901 10.3389/fnmol.2020.594220 PMC7773664

[18] Cartaud A, Strochlic L, Guerra M, et al MuSK is required for anchoring acetylcholinesterase at the neuromuscular junction. J Cell Biol. 2004;165(4):505–515. 15159418 10.1083/jcb.200307164 PMC2172359

[19] Sigoillot SM, Bourgeois F, Lambergeon M, Strochlic L, Legay C. Colq controls postsynaptic differentiation at the neuromuscular junction. J Neurosci. 2010;30(1):13–23. 20053883 10.1523/JNEUROSCI.4374-09.2010 PMC6632527

[20] Sigoillot SM, Bourgeois F, Karmouch J, et al Neuromuscular junction immaturity and muscle atrophy are hallmarks of the ColQ-deficient mouse, a model of congenital myasthenic syndrome with acetylcholinesterase deficiency. FASEB J. 2016;30(6):2382–2399. 26993635 10.1096/fj.201500162

[21] Feng G, Krejci E, Molgo J, Cunningham JM, Massoulié J, Sanes JR. Genetic analysis of collagen Q: Roles in acetylcholinesterase and butyrylcholinesterase assembly and in synaptic structure and function. J Cell Biol. 1999;144(6):1349–1360. 10087275 10.1083/jcb.144.6.1349 PMC2150590

[22] Donger C, Krejci E, Serradell AP, et al Mutation in the human acetylcholinesterase-associated collagen gene, COLQ, is responsible for congenital myasthenic syndrome with end-plate acetylcholinesterase deficiency (Type Ic). Am J Hum Genet. 1998;63(4):967–975. 9758617 10.1086/302059 PMC1377491

[23] Ohno K, Brengman J, Tsujino A, Engel AG. Human endplate acetylcholinesterase deficiency caused by mutations in the collagen-like tail subunit (ColQ) of the asymmetric enzyme. Proc Natl Acad Sci U S A. 1998;95(16):9654–9659. 9689136 10.1073/pnas.95.16.9654 PMC21394

[24] McMacken GM, Spendiff S, Whittaker RG, et al Salbutamol modifies the neuromuscular junction in a mouse model of ColQ myasthenic syndrome. Hum Mol Genet. 2019;28(14):2339–2351. 31220253 10.1093/hmg/ddz059 PMC6606850

[25] Otsuka K, Ito M, Ohkawara B, et al Collagen Q and anti-MuSK autoantibody competitively suppress agrin/LRP4/MuSK signaling. Sci Rep. 2015;5:13928. 26355076 10.1038/srep13928 PMC4564764

[26] Theuriet J, Masingue M, Behin A, et al Congenital myasthenic syndromes in adults: Clinical features, diagnosis and long-term prognosis. Brain. 2024;147(11):3849–3862. 38696726 10.1093/brain/awae124 PMC11531845

[27] Willmann R, Dubach J, Chen K. Developing standard procedures for pre-clinical efficacy studies in mouse models of spinal muscular atrophy. Neuromuscul Disord. 2011;21(1):74–77. 21115346 10.1016/j.nmd.2010.09.014

[28] Mill L, Mürdter L. *Mira Vision*. Accessed 29 June 2023. https://www.mira.vision/

[29] Jones RA, Reich CD, Dissanayake KN, et al NMJ-morph reveals principal components of synaptic morphology influencing structure-function relationships at the neuromuscular junction. Open Biol. 2016;6(12):160240. 27927794 10.1098/rsob.160240 PMC5204123

[30] Maselli RA, Fernandez JM, Arredondo J, et al LG2 agrin mutation causing severe congenital myasthenic syndrome mimics functional characteristics of non-neural (z-) agrin. Hum Genet. 2012;131(7):1123–1135. 22205389 10.1007/s00439-011-1132-4 PMC4795461

[31] Nicole S, Chaouch A, Torbergsen T, et al Agrin mutations lead to a congenital myasthenic syndrome with distal muscle weakness and atrophy. Brain. 2014;137(Pt 9):2429–2443. 24951643 10.1093/brain/awu160

[32] Huzé C, Bauché S, Richard P, et al Identification of an agrin mutation that causes congenital myasthenia and affects synapse function. Am J Hum Genet. 2009;85(2):155–167. 19631309 10.1016/j.ajhg.2009.06.015 PMC2725239

[33] Thompson R, Bonne G, Missier P, Lochmüller H. Targeted therapies for congenital myasthenic syndromes: Systematic review and steps towards a treatabolome. Emerg Top Life Sci. 2019;3(1):19–37. 30931400 10.1042/ETLS20180100 PMC6436731

[34] Schiaffino S, Reggiani C. Fiber types in mammalian skeletal muscles. Physiol Rev. 2011;91(4):1447–1531. 22013216 10.1152/physrev.00031.2010

[35] Oury J, Gamallo-Lana B, Santana L, et al Agonist antibody to MuSK protects mice from MuSK myasthenia gravis. Proc Natl Acad Sci U S A. 2024;121(39):e2408324121. 39288173 10.1073/pnas.2408324121 PMC11441477

[36] Cantor S, Zhang W, Delestrée N, Remédio L, Mentis GZ, Burden SJ. Preserving neuromuscular synapses in ALS by stimulating MuSK with a therapeutic agonist antibody. Elife. 2018;7:e34375. 29460776 10.7554/eLife.34375 PMC5837562

[37] Sengupta-Ghosh A, Dominguez SL, Xie L, et al Muscle specific kinase (MuSK) activation preserves neuromuscular junctions in the diaphragm but is not sufficient to provide a functional benefit in the SOD1G93A mouse model of ALS. Neurobiol Dis. 2019;124:340–352. 30528255 10.1016/j.nbd.2018.12.002

[38] Sun S, Shen Y, Zhang X, et al The MuSK agonist antibody protects the neuromuscular junction and extends the lifespan in C9orf72-ALS mice. Mol Ther. 2024;32(7):2176–2189. 38734896 10.1016/j.ymthe.2024.05.016 PMC11286808

[39] Feng Z, Lam S, Tenn EMS, et al Activation of Muscle-Specific Kinase (MuSK) reduces neuromuscular defects in the Delta7 mouse model of Spinal Muscular Atrophy (SMA). Int J Mol Sci. 2021;22(15):8015. 34360794 10.3390/ijms22158015 PMC8348537

[40] Valenzuela DM, Stitt TN, DiStefano PS, et al Receptor tyrosine kinase specific for the skeletal muscle lineage: Expression in embryonic muscle, at the neuromuscular junction, and after injury. Neuron. 1995;15(3):573–584. 7546737 10.1016/0896-6273(95)90146-9

[41] Jacquier A, Risson V, Simonet T, et al Severe congenital myasthenic syndromes caused by agrin mutations affecting secretion by motoneurons. Acta Neuropathol. 2022;144(4):707–731. 35948834 10.1007/s00401-022-02475-8 PMC9468088

[42] Kim N, Burden SJ. MuSK controls where motor axons grow and form synapses. Nat Neurosci. 2008;11(1):19–27. 18084289 10.1038/nn2026 PMC2923649

[43] Uyen Dao TM, Barbeau S, Messéant J, et al The collagen ColQ binds to LRP4 and regulates the activation of the Muscle-Specific Kinase-LRP4 receptor complex by agrin at the neuromuscular junction. J Biol Chem. 2023;299(8):104962. 37356721 10.1016/j.jbc.2023.104962 PMC10382678

[44] Mihaylova V, Müller JS, Vilchez JJ, et al Clinical and molecular genetic findings in COLQ-mutant congenital myasthenic syndromes. Brain. 2008;131(Pt 3):747–759. 18180250 10.1093/brain/awm325

[45] Ohkawara B, Cabrera-Serrano M, Nakata T, et al LRP4 third β-propeller domain mutations cause novel congenital myasthenia by compromising agrin-mediated MuSK signaling in a position-specific manner. Hum Mol Genet. 2014;23(7):1856–1868. 24234652 10.1093/hmg/ddt578 PMC3943522

[46] Chevessier F, Girard E, Molgó J, et al A mouse model for congenital myasthenic syndrome due to MuSK mutations reveals defects in structure and function of neuromuscular junctions. Hum Mol Genet. 2008;17(22):3577–3595. 18718936 10.1093/hmg/ddn251

[47] Maselli RA, Arredondo J, Cagney O, et al Mutations in MUSK causing congenital myasthenic syndrome impair MuSK-Dok-7 interaction. Hum Mol Genet. 2010;19(12):2370–2379. 20371544 10.1093/hmg/ddq110 PMC2876883

[48] Webster RG, Vanhaesebrouck AE, Maxwell SE, et al Effect of salbutamol on neuromuscular junction function and structure in a mouse model of DOK7 congenital myasthenia. Hum Mol Genet. 2020;29(14):2325–2336. 32543656 10.1093/hmg/ddaa116 PMC7424765

[49] Clausen L, Cossins J, Beeson D. Beta-2 adrenergic receptor agonists enhance AChR clustering in C2C12 myotubes: Implications for therapy of myasthenic disorders. J Neuromuscul Dis. 2018;5(2):231–240. 29865088 10.3233/JND-170293 PMC6004912

[50] Lim JL, Jensen SM, Plomp JJ, et al Patient-specific therapeutic benefit of MuSK agonist antibody ARGX-119 in MuSK myasthenia gravis passive transfer models. iScience. 2025;28(2):111684. 39898046 10.1016/j.isci.2024.111684 PMC11783450

