# Supplementary material for: Gene-specific response to muscle specific kinase agonist antibody in the treatment of congenital myasthenic syndromes
Source: Brain Commun. 2026 May 14;8(3):fcag115. doi: 10.1093/braincomms/fcag115 (PMC13174946; doi:10.1093/braincomms/fcag115)
Supplement: fcag115_Supplementary_Data [file fcag115_supplementary_data.docx]

**Supplementary material**

# Supplementary figures


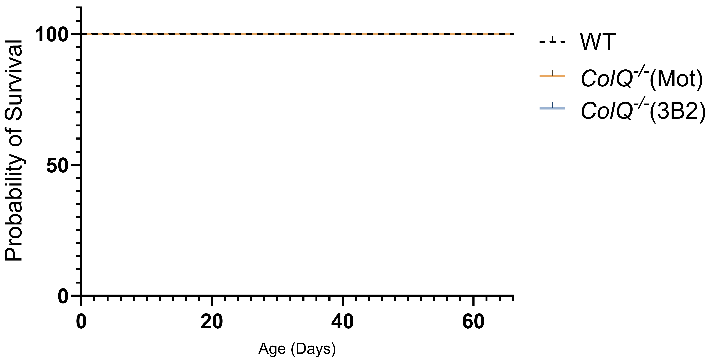


**Supplementary Figure 1. Survival of *ColQ^-/-^* mice.** Kaplan-Meier curve showing **a**ll animals survived to the end of the study at P66. *x*^2^ = 0.00 WT n=10, *ColQ*^-/-^ (Mot) n=10, *ColQ*^-/-^ (3B2) n=12

**
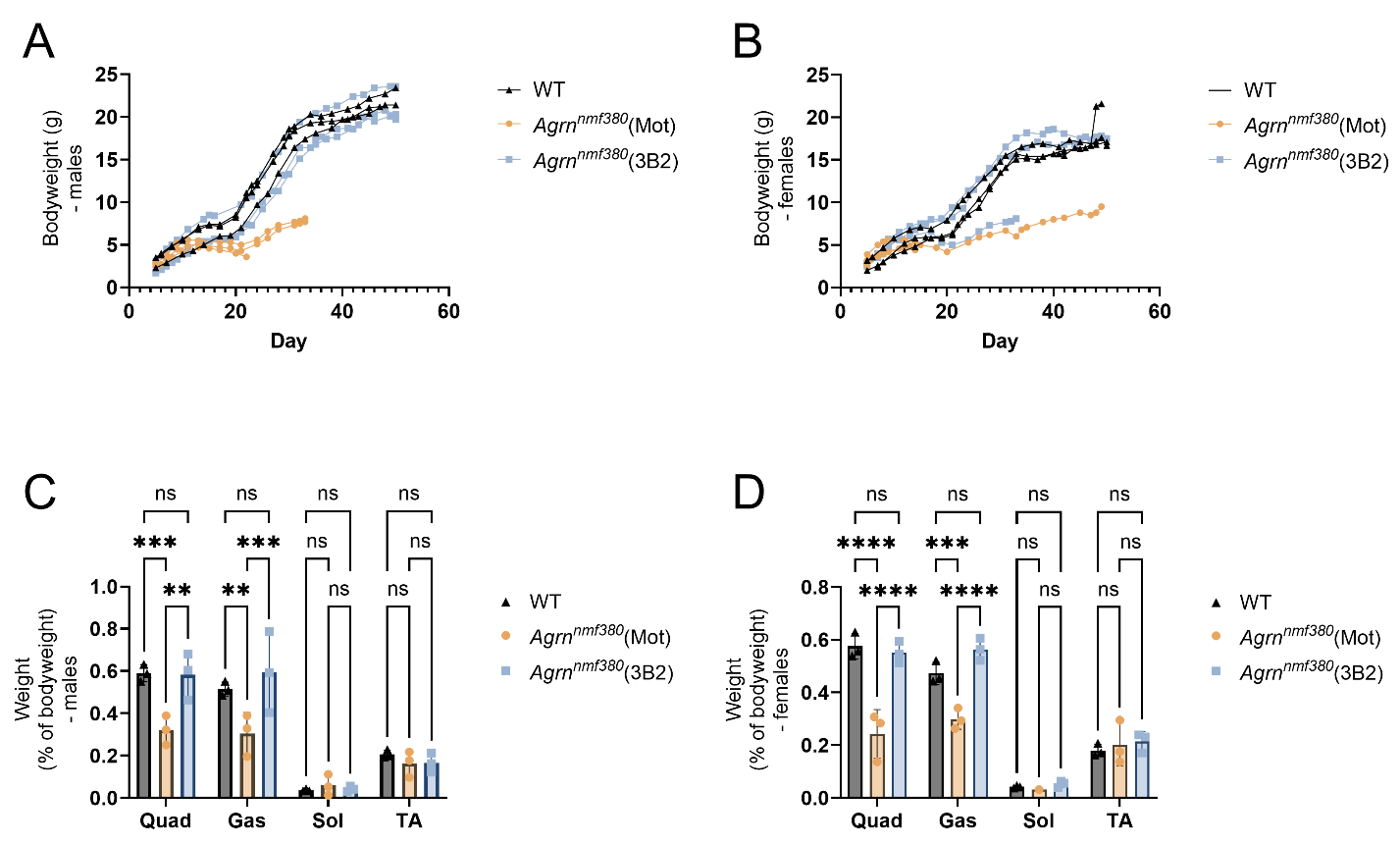
**

**Supplementary Figure 2. Bodyweight and muscle weights of male and female *Agrn^nmf380^* and WT mice. (A)** 3B2 treatment rescued the bodyweight of male *Agrn^nmf380^* mice *F*(2,120) = 36.61 and **(B)** female *Agrn^nmf380^* mice. *F*(2,110) = 27.39 **(C)** 3B2 treatment rescued the muscle weights of male *Agrn^nmf380^* mice *F*(2,24) = 10.81 and **(D)** female *Agrn^nmf380^* mice. *F*(2,22) = 24.44 **(A)** & **(B)** Each data point represents each mouse. 2-Way mixed-effects analysis with Tukey’s multiple comparisons correction. **(C)** & **(D)** Each data point represents each mouse. Graphs show mean ± sd. 2-Way ANOVA with Tukey’s multiple comparisons correction. n=3 animals per group. **p<0.005, ***p<0.001, ****p<0.0001, ns=non-significant.


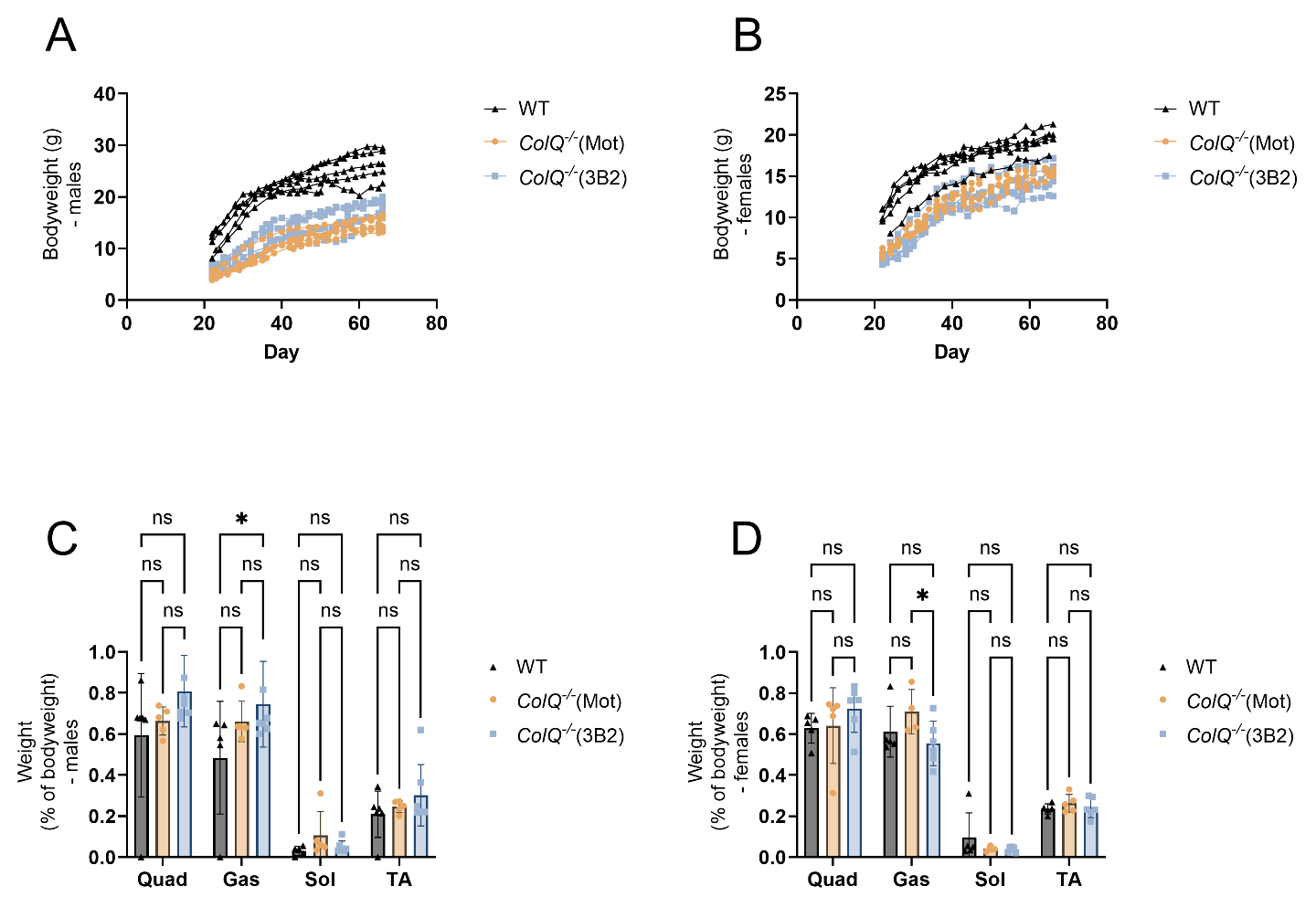


**Supplementary Figure 3. Bodyweight and muscle weights of male and female *ColQ^-/-^* and WT mice. (A)** 3B2 treatment did not improve the bodyweight of male *ColQ*^-/-^ mice *F*(2,291) = 985.8 **(B)** and female *ColQ*^-/-^ mice. *F*(2,283) = 517.6 **(C)** Muscle weights of male *ColQ*^-/-^ and WT mice *F*(2,58) = 5.06 and **(D)** female *ColQ*^-/-^ and WT mice. *F*(2,51) = 0.36 **(A)** & **(B)** Each data point represents each mouse. 2-Way mixed-effects analysis with Tukey’s multiple comparisons correction. **(C)** & **(D)** Each data point represents each mouse. Graphs show mean ± sd. 2-Way ANOVA with Tukey’s multiple comparisons correction. WT n=5, *ColQ*^-/-^ (Mot) n=5, *ColQ*^-/-^ (3B2) n=6. *p<0.05, ns=non-significant.


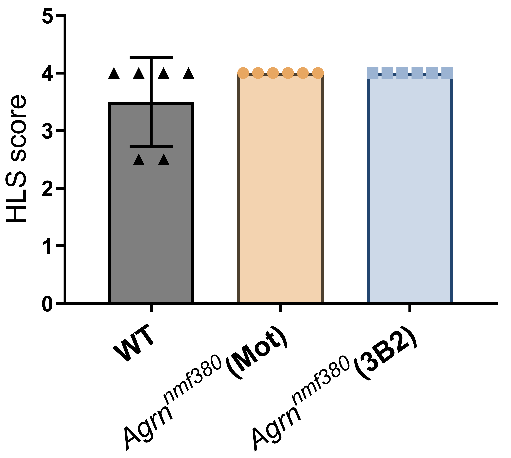


**Supplementary Figure 4. Hindlimb suspension score (HLS) in *Agrn^nmf380^* and WT mice.** There were no differences in the HLS score between groups. *F*(2,15) = 2.50 Each data point represents each mouse. Graphs show mean ± sd. 1-Way ANOVA with Tukey’s multiple comparisons correction. WT n=6, *Agrn^nmf380^* (Mot) n=6, *Agrn^nmf380^* (3B2) n=6.


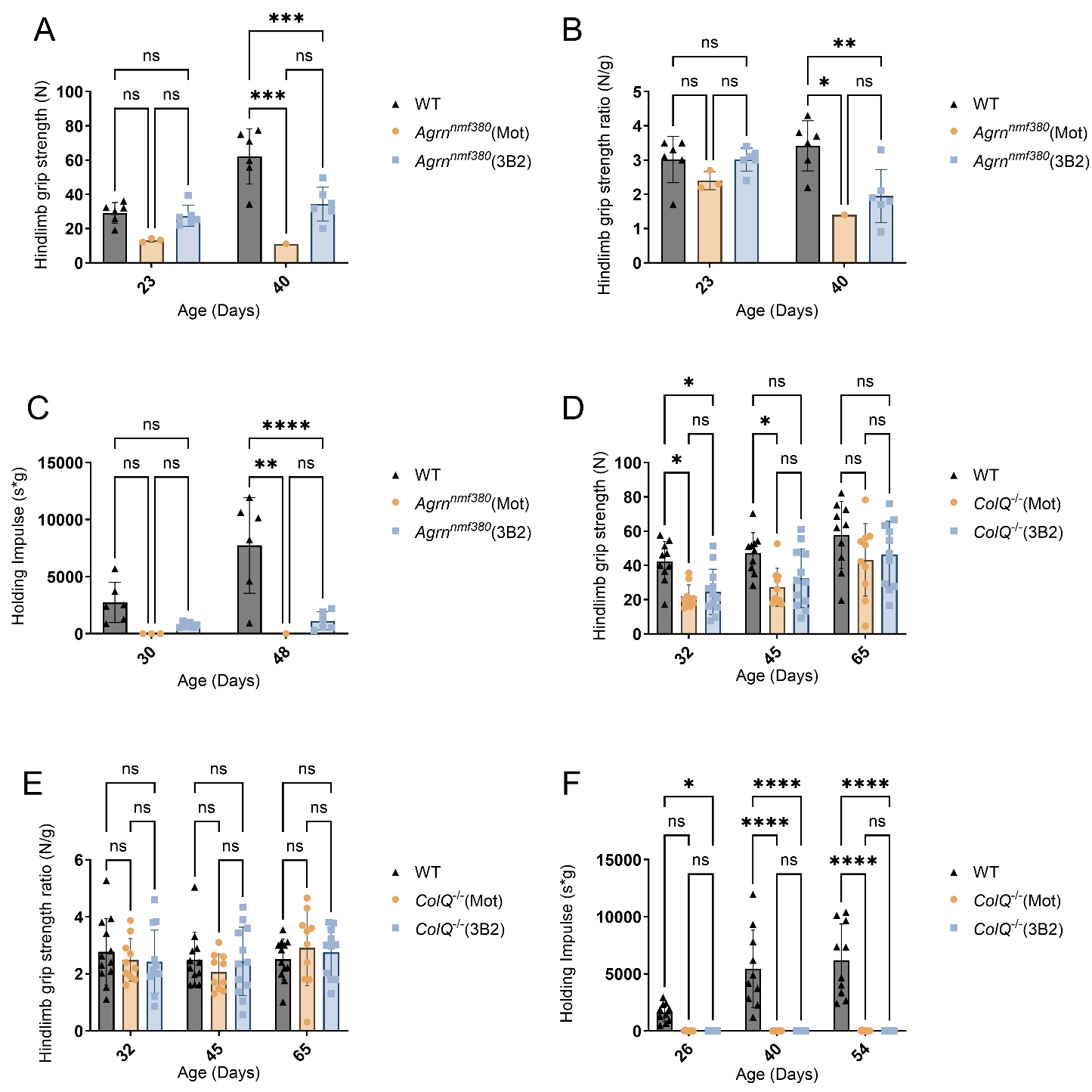


**Supplementary Figure 5. Strength assessments in *Agrn^nmf380^* and *ColQ^-/-^* animals (A)** Hindlimb grip strength revealed differences between WT and *Agrn^nmf380^* mice at P40. *F*(2,22) = 15.95 **(B)** There were no differences in hindlimb grip strength ratio between *Agrn^nmf380^* animals treated with Mot or 3B2. *F*(2,22) = 7.12 **(C)** WT animals did not have a higher holding impulse than *Agrn^nmf380^* (Mot) or *Agrn^nmf380^* (3B2) animals until P48. There was no significant difference in average holding impulse with 3B2 treatment in *Agrn^nmf380^* mice. *F*(2,22) = 12.47 **(D)** WT animals had greater hindlimb strength than *ColQ^-/-^* animals, *F*(2,86) = 12.00 (**E**) though these differences disappeared when normalized for body weight and no improvements were seen with 3B2 treatment. *F*(2,92) = 0.09 **(F)** A similar pattern was observed in the holding impulse of the inverted screen test. *F*(2,87) = 87.69 Each data point represents each mouse. Graphs show mean ± sd. 2-Way ANOVA with Tukey’s multiple comparisons correction. At P23 & 31, WT n=6, *Agrn^nmf380^* (Mot) n=3, *Agrn^nmf380^* (3B2) n=6, at P42 & P48 WT n=6, *Agrn^nmf380^* (Mot) n=1, *Agrn^nmf380^* (3B2) n=6. WT n=10, *ColQ*^-/-^ (Mot) n=10, *ColQ*^-/-^ (3B2) n=12. *p<0.05, **p<0.005, ***p<0.001, ****p<0.0001, ns=non-significant.

**
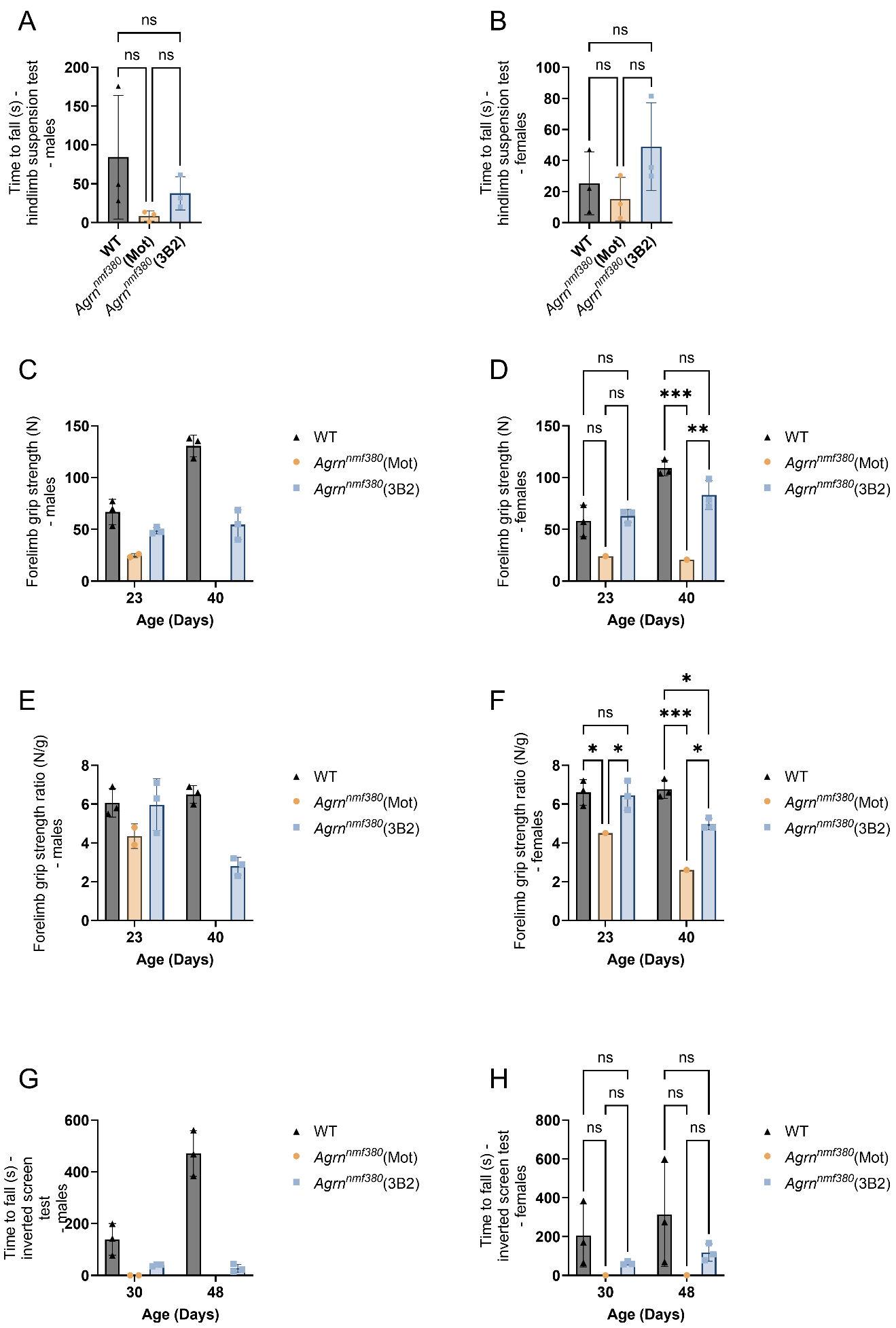
**

**Supplementary Figure 6. Motor behavioural testing of male and female *Agrn^nmf380^* and WT mice. (A)** Time to fall in seconds of hindlimb suspension test of male *Agrn^nmf380^* and WT mice *F*(2,6) = 1.92, and **(B)** female *Agrn^nmf380^* and WT mice. *F*(2,6) = 1.93 **(C)** Forelimb grip strength of male *Agrn^nmf380^* and WT mice, and **(D)** female *Agrn^nmf380^* and WT mice. *F*(2,4) = 17.53 **(E)** Forelimb grip strength normalized to bodyweight of male *Agrn^nmf380^* and WT mice, and **(F)** female *Agrn^nmf380^* and WT mice. *F*(2,8) = 22.87 **(G)** Time to fall in seconds of inverted screen test of male *Agrn^nmf380^* and WT mice, and **(H)** female *Agrn^nmf380^* and WT mice. *F*(2,4) = 2.37 Each data point represents each mouse. Graphs show mean ± sd. **(A)** & **(B)** 1-Way ANOVA with Tukey’s multiple comparisons correction. **(D)**, **(F)** & **(H)** 2-Way ANOVA with Tukey’s multiple comparisons correction. n=3 animals per group. *p<0.05, **p<0.005, ***p<0.001, ns=non-significant.

**
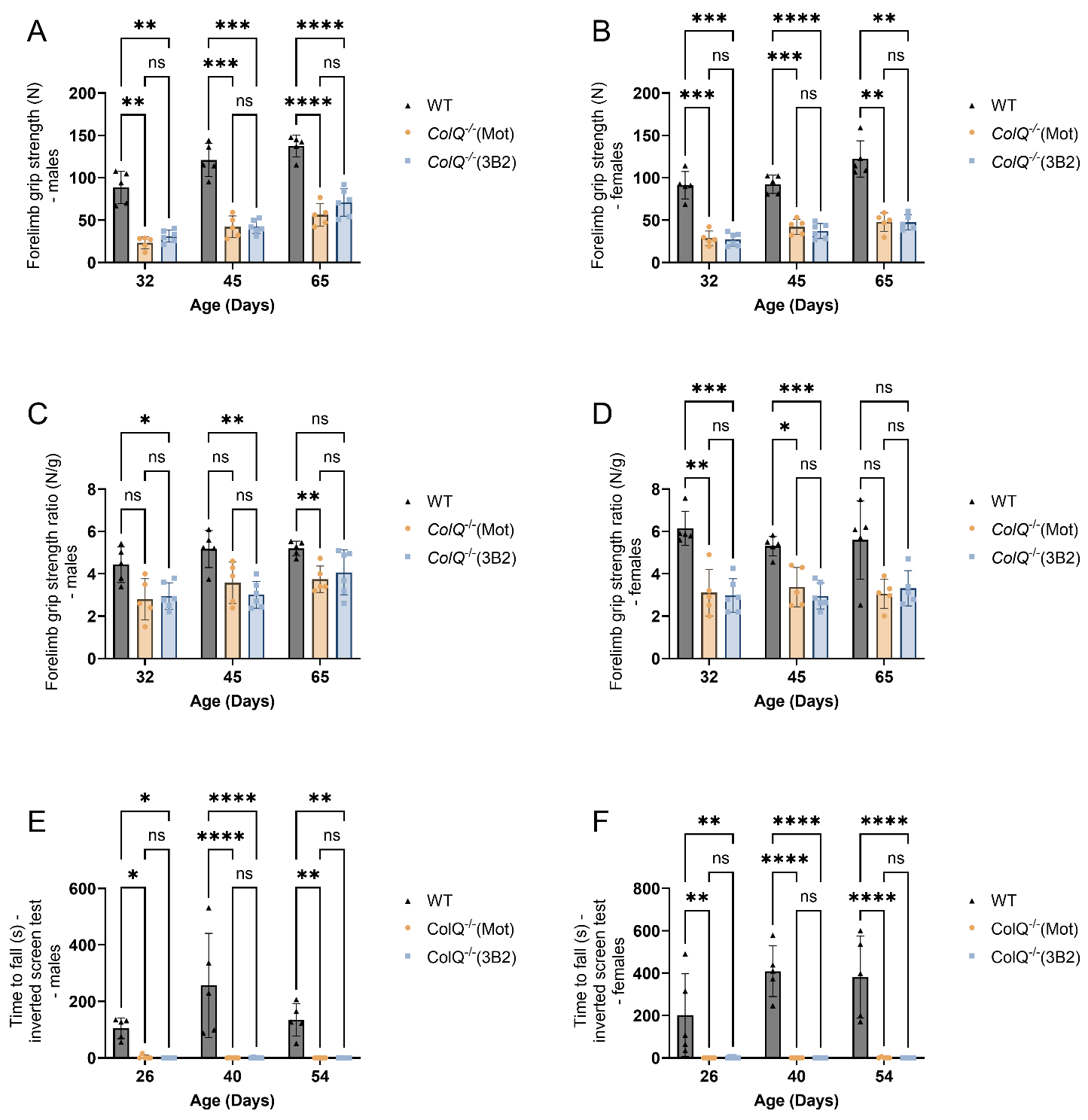
**

**Supplementary Figure 7. Motor behavioural testing of male and female *ColQ^-/-^* and WT mice. (A)** Forelimb grip strength of male *ColQ^-/-^* and WT mice *F*(2,13) = 107.00, and **(B)** female *ColQ^-/-^* and WT mice. *F*(2,13) = 111.00 **(C)** Forelimb grip strength normalized to bodyweight of male *ColQ^-/-^* and WT mice *F*(2,13) = 16.73, and **(D)** female *ColQ^-/-^* and WT mice. *F*(2,38) = 36.05 **(E)** Time to fall in seconds of inverted screen test of male *ColQ^-/-^* and WT mice *F*(2,39) = 35.15, and **(F)** female *ColQ^-/-^* and WT mice. *F*(2,39) = 61.17 Each data point represents each mouse. Graphs show mean ± sd. 2-Way ANOVA with Tukey’s multiple comparisons correction. WT n=5, *ColQ*^-/-^ (Mot) n=5, *ColQ*^-/-^ (3B2) n=6. *p<0.05, **p<0.005, ***p<0.001, ****p<0.0001, ns=non-significant.


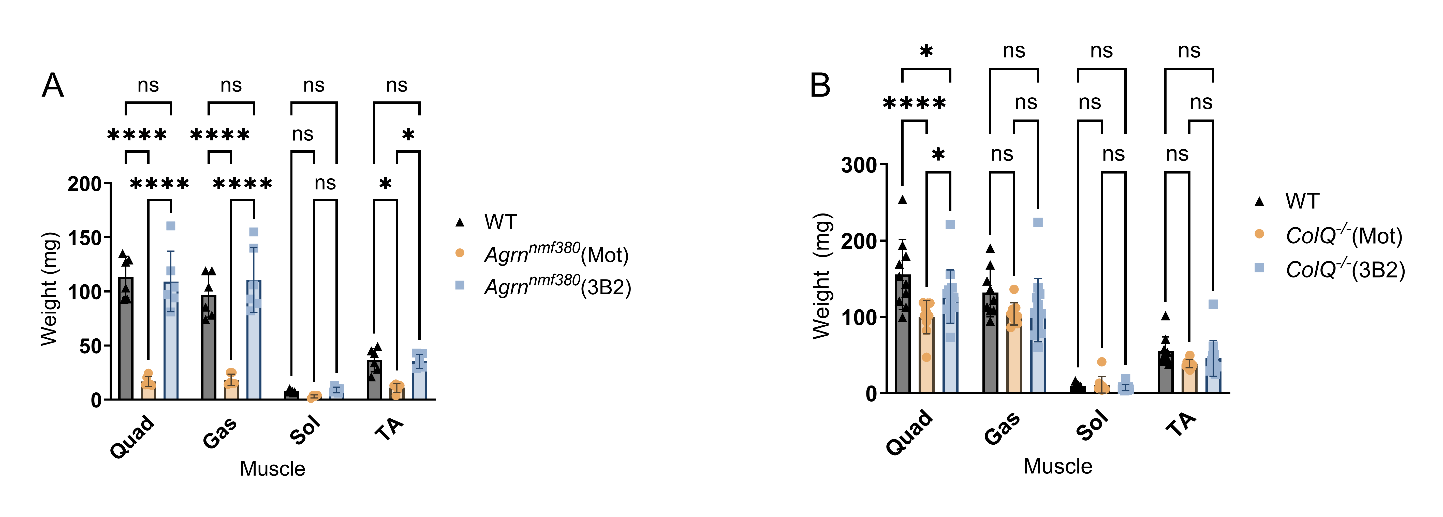


**Supplementary Figure 8. Raw values muscle weight in WT, 3B2 treated and untreated animals.** **(A)** 3B2 treatment fully rescued quadriceps (Quad), gastrocnemius (Gas), and tibialis anterior (TA) muscle weights of *Agrn^nmf380^* mice back to WT levels. There were no differences in soleus (Sol) muscle weights. WT n=6, *Agrn^nmf380^* (Mot) n=6, *Agrn^nmf380^* (3B2) n=6. *F*(2,58) = 88.25 **(B)** *ColQ^-/-^* (3B2) mice had heavier quad muscles compared to *ColQ^-/-^* (Mot) mice. *F*(2,117) = 8.73 WT n=10, *ColQ*^-/-^ (Mot) n=10, *ColQ*^/-^ (3B2) n=12. Each data point represents each mouse. Graphs show mean ± sd. 2-Way ANOVA with Tukey’s multiple comparisons correction. *p<0.05, ****p<0.0001, ns=non-significant.


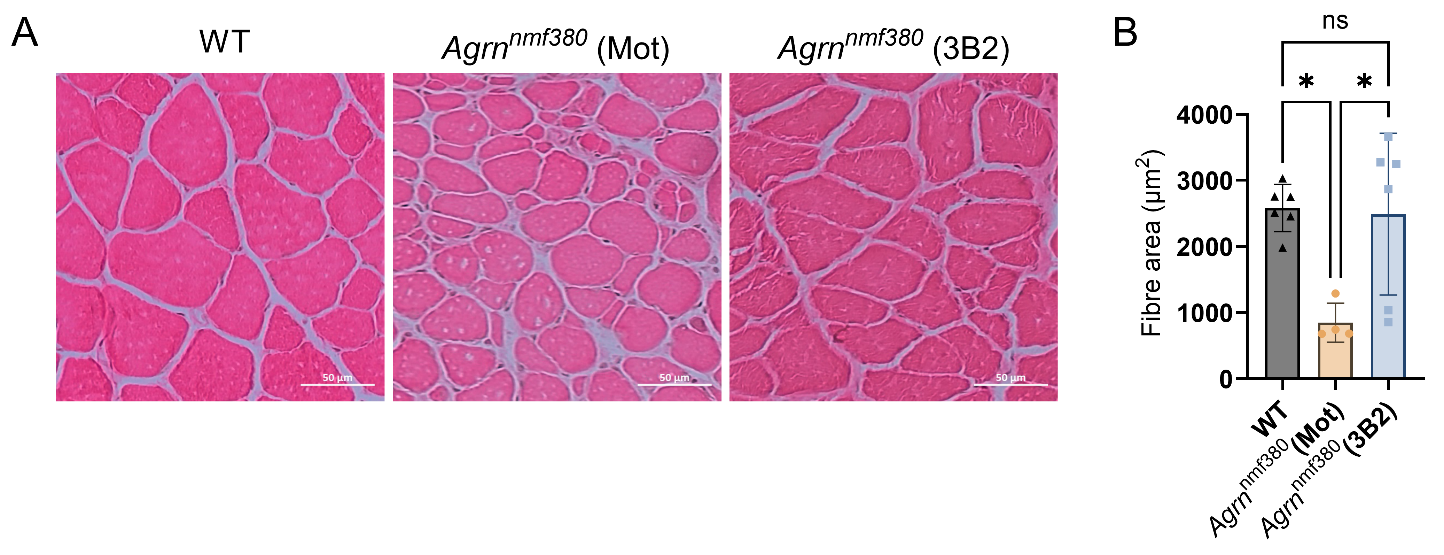


**Supplementary Figure 9. Quadriceps fibre size in *Agrn^nmf380^* mice. (A)** Haematoxylin and eosin was used to label muscle cross sections from WT (left), *Agrn^nmf380^* (Mot) (middle), and *Agrn^nmf380^* (3B2) (right) mice quadriceps. **(B)** *Agrn^nmf380^* (Mot) mice had significantly decreased myofiber size compared to both WT mice and *Agrn^nmf380^* (3B2) mice. *F*(2,13) = 6.66 Each data point represents each mouse. Graphs show mean ± sd. 1-Way ANOVA with Tukey’s multiple comparisons correction. WT n=6, *Agrn^nmf380^* (Mot) n=4, *Agrn^nmf380^* (3B2) n=6. *p<0.05, ns=non-significant.


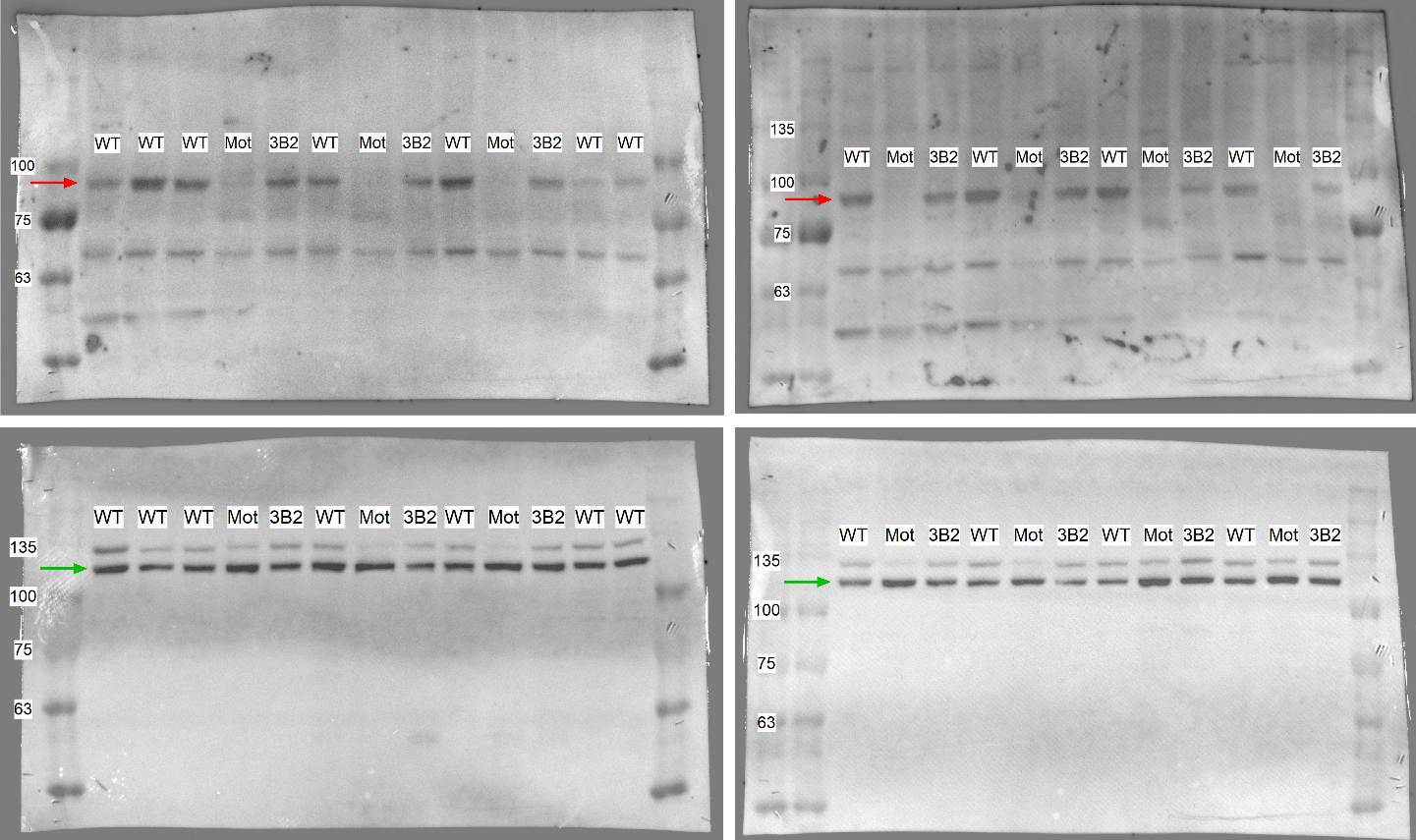


**Supplementary Figure 10. Full Western Blot images used to quantify pMuSK in WT and *Agrn^nmf380^* mice.** Bands were quantified and normalised to vinculin loading control. Some samples were run on more than one blot so that samples could be normalised across different blots. pMuSK is identified (top gels, red arrow) at 93kD. Loading control is vinculin (bottom gels, green arrow) at 120kD. These uncropped blots correspond to Figure 5F in the main manuscript.

**
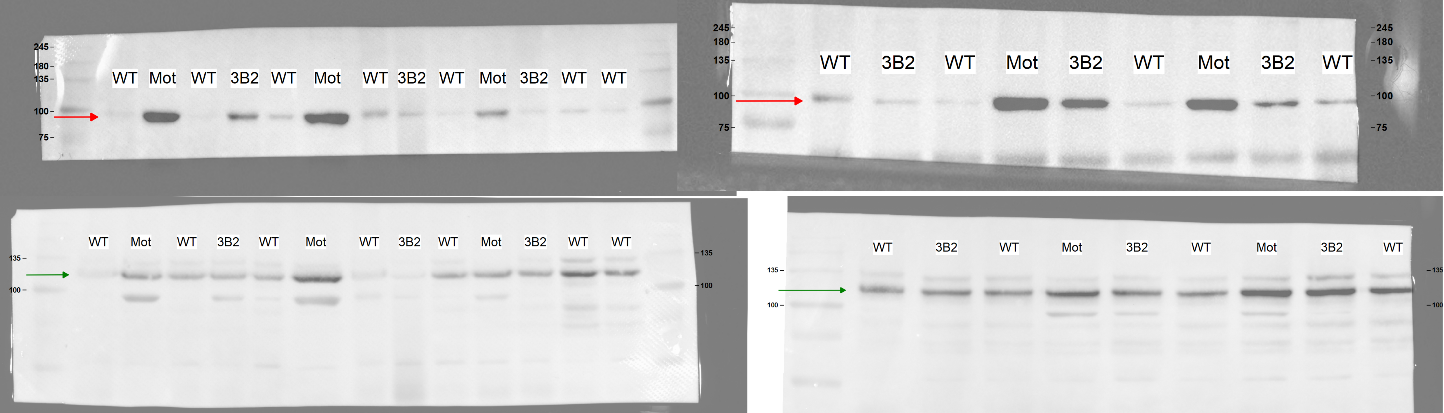
**

**Supplementary Figure 11. Full Western Blot images used to quantify MuSK in WT and *Agrn^nmf380^* mice.** Bands were quantified and normalised to vinculin loading control. Some samples were run on more than one blot so that samples could be normalised across different blots. Thick ladder band is 75kDa and MuSK is identified (top gels, red arrow) at 95kD. Loading control is vinculin (bottom gels, red arrow) at 120kD. These uncropped blots correspond to Figure 5E in the main manuscript.


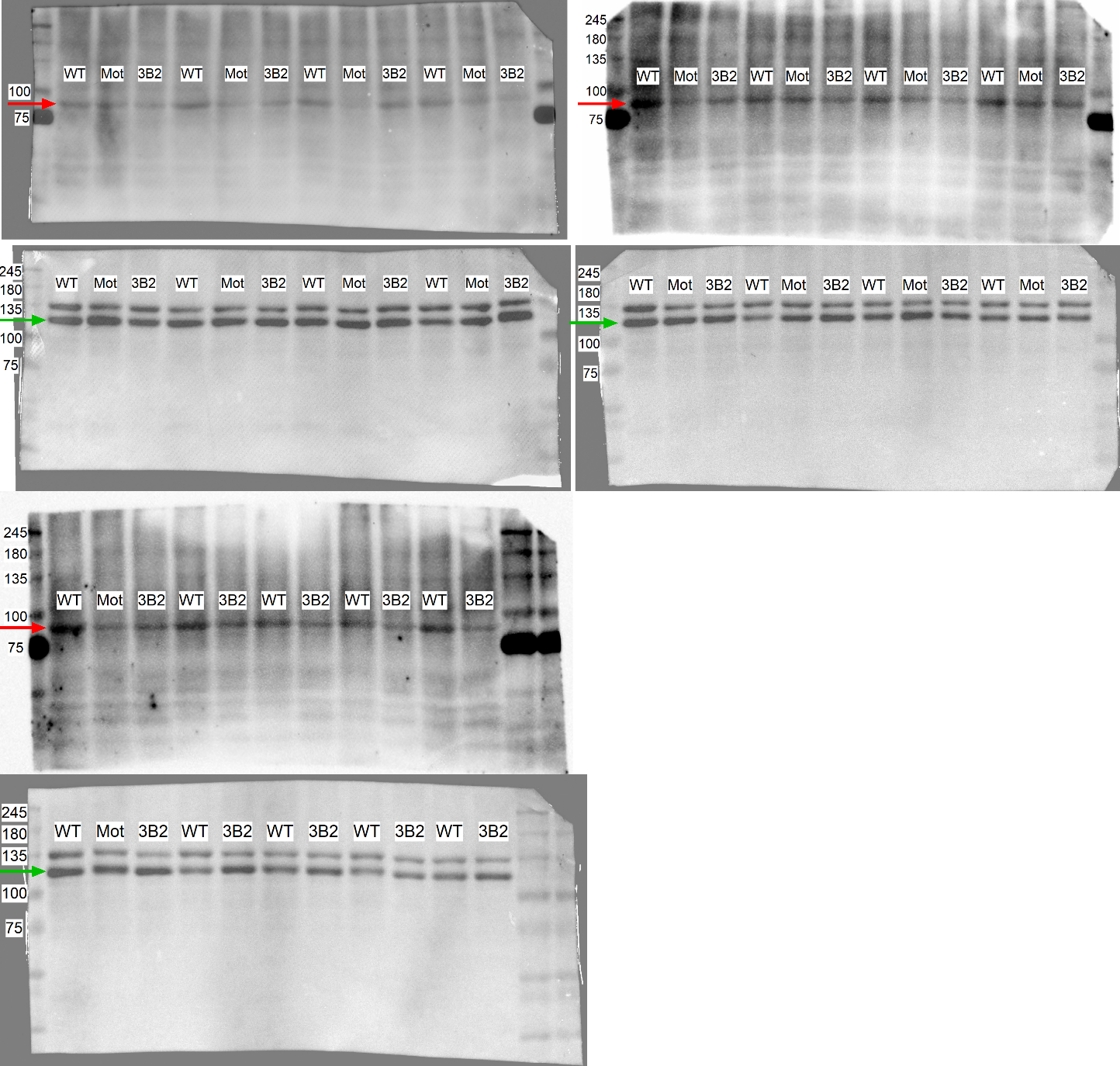


**Supplementary Figure 12. Full Western blot images used to quantify pMuSK in WT and *ColQ^-/-^* mice.** Bands were quantified and normalised to vinculin loading control. Some samples were run on more than one blot so that samples could be normalised across different blots. pMuSK is identified (top gels, red arrow) at 93kD. Loading control is vinculin (bottom gels, green arrow) at 120kD. These uncropped blots correspond to Figure 6F in the main manuscript.

**
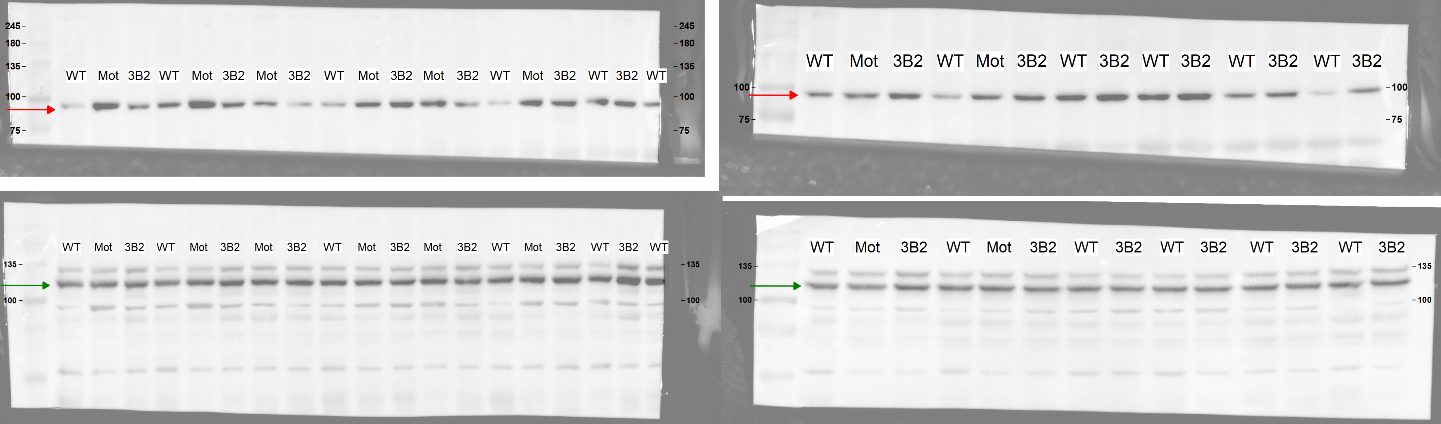
Supplementary Figure 13. Full Western Blot images used to quantify MuSK in WT and** ***ColQ^-/-^* mice.** Bands were quantified and normalised to vinculin loading control. Some samples were run on more than one blot so that samples could be normalised across different blots. pMuSK is identified (top gels, red arrow) at 93kD. Loading control is vinculin (bottom gels, green arrow) at 120kD. These uncropped blots correspond to Figure 6E in the main manuscript.


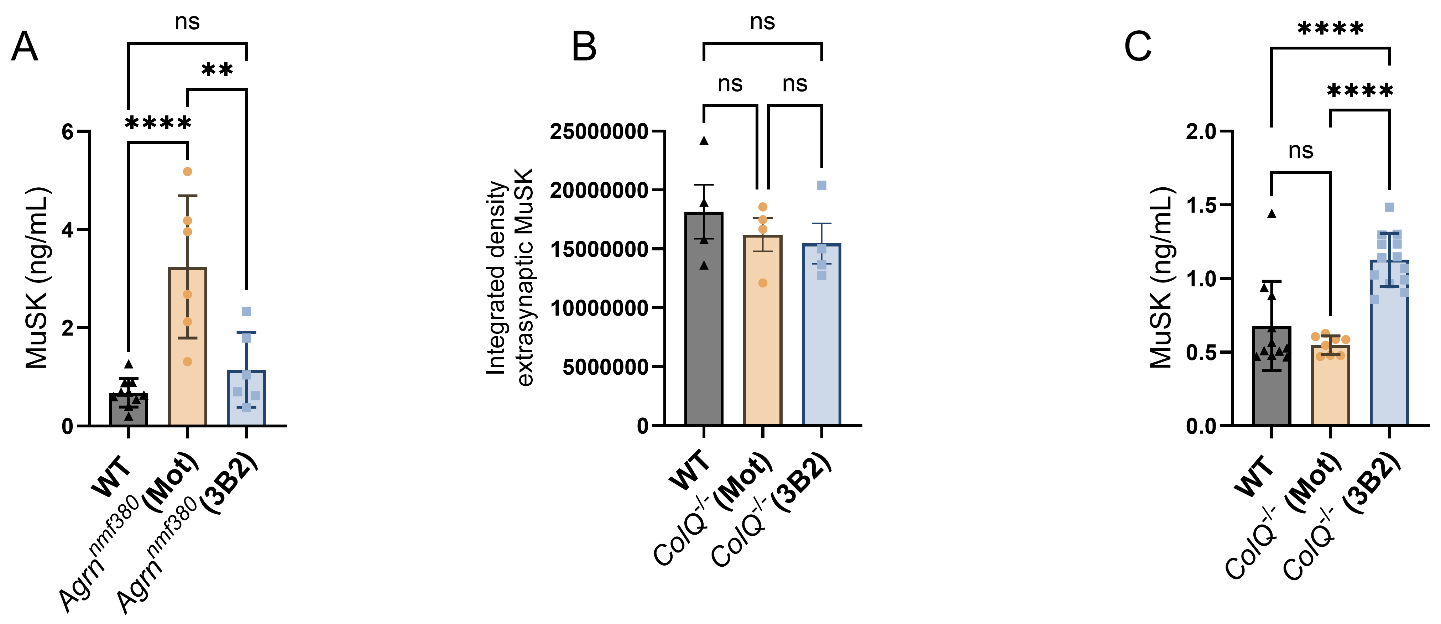
**Supplementary Figure 14. MuSK in *Agrn^nmf380^* and *ColQ*^-/-^ mice. (A)** When quantified with an ELISA, *Agrn^nmf380^* (Mot) mice had higher total MuSK levels in TA muscle lysate. *F*(2,19) = 17.24 **(B)** Integrated density of extrasynaptic MuSK was determined through immunostaining and no differences were found between WT, *ColQ*^-/-^ (Mot) and *ColQ*^-/-^ (3B2) mice. *F*(2,9) = 0.57 (C) ELISA results show that *ColQ*^-/-^ (3B2) mice had increased levels of MuSK. *F*(2,29) = 22.14 Each data point represents each mouse. Graphs show mean ± sd. 1-Way ANOVA with Tukey’s multiple comparisons correction. **(A)** WT n=10, *Agrn^nmf380^* (Mot) n=6, *Agrn^nmf380^* (3B2) n=6 **(B)** n=4 animals per group. **(C)** WT n=11, *ColQ*^-/-^ (Mot) n=8, *ColQ*^-/-^ (3B2) n=13 **p<0.005, ****p<0.0001, ns=non-significant.

# Supplementary tables

**Supplementary Table 1: Treatment protocol showing timings and dosages for experimental, control, and WT mice.**

| **Model** | **N** | **Genotype** | **Treatment** | **Dose Level (mg/kg)** | **Route** | **Regimen** |
| --- | --- | --- | --- | --- | --- | --- |
| AGRN | 6 | *Agrnnmf380* | 3B2 | 20 mg/kg at P5, 10mg/kg at P15 and P35 | IP | P5, P15, P35 |
|  | 6 |  | Mota |  |  |  |
|  | 6 | Wild type | None | N/A | N/A | N/A |
| ColQ | 12 | *ColQ*^-/-^ mice | 3B2 | 20 mg/kg at P22, followed by weekly 10 mg/kg | IP | P22, P29, P36, P43, P50 and P57 |
|  | 10 |  | Mota |  |  |  |
|  | 10 | Wild type | None | N/A | N/A | N/A |

**Supplementary Table 2: Serum concentrations of 3B2 in WT and *Agrn^nmf380^* mice**.

| **Group** | **Animal ID** | **Dosing** | **Sample point** | **[3B2] (µg/mL)** | **Sample point** | **[3B2] (µg/mL)** |
| --- | --- | --- | --- | --- | --- | --- |
| WT | T2M1 | NA | P35 | <LLOQ | P50 | <LLOQ |
|  | T2F2 |  |  | NS |  | <LLOQ |
|  | Y1M2 |  |  | NS |  | <LLOQ |
|  | Y1M3 |  |  | NS |  | <LLOQ |
|  | Y1M4 |  |  | NS |  | <LLOQ |
|  | Y1M6 |  |  | NS |  | <LLOQ |
|  | Y1F1 |  |  | NS |  | <LLOQ |
|  | AK3F1 |  |  | <LLOQ |  | <LLOQ |
|  | AK3M2 |  |  | <LLOQ |  | <LLOQ |
|  | AK3M3 |  |  | <LLOQ |  | <LLOQ |
| *Agrnnmf380*  (Mot) | X1M3 | 20 mg/kg at P5,  10mg/kg at P15 and  P35 | P35 | NS | P50 | <LLOQ |
|  | AH3F3 |  |  | NS |  | NS |
|  | AH3F5 |  |  | NS |  | NS |
|  | AJ2F1 |  |  | NS |  | <LLOQ |
|  | AJ5M2 |  |  | NS |  | <LLOQ |
|  | AJ5M4 |  |  | NS |  | <LLOQ |
| *Agrnnmf380*  (3B2) | AG3F1 | 20 mg/kg at P5,  10mg/kg at P15 and  P35 | P35 | 12.9 | P50 | 44.05 |
|  | AJ5F3 |  |  | 3.91 |  | NS |
|  | AJ5F5 |  |  | 7.79 |  | 4.00 |
|  | AL2M4 |  |  | 10.27 |  | 15.20 |
|  | AG3M2 |  |  | 12.54 |  | 36.80 |
|  | AG3M4 |  |  | 1.94 |  | 39.74 |

ELISAs were performed on serum to determine the levels of 3B2. As expected, levels of 3B2 in the WT and *Agrn^nmf380^* (Mot) mice were undetectable, but detectable in the *Agrn^nmf380^* (3B2) animals at both P35 and P50. Furthermore, there was a significant increase in 3B2 serum levels in the *Agrn^nmf380^* (3B2) mice from P35 to P50, demonstrating the accumulation of the compound in the blood. **NS** no samples taken (Animal care guidelines dictate a minimum body weight that must be reached before blood collection can be performed), **LLOQ** Lower limit of quantification (3B2 0.1µg/mL), **NA** not applicable.

**Supplementary Table 3: Serum concentrations of 3B2 in WT and *ColQ^-/-^* mice.**

| **Group** | **Animal ID** | **Dosing** | **Sample point** | **[3B2] (µg/mL)** | **Sample point** | **[3B2] (µg/mL)** | **Sample point** | **[3B2](µg/mL)** |
| --- | --- | --- | --- | --- | --- | --- | --- | --- |
| WT | AW2F3 | NA | P29 | NS | P43 | NES | P66 | <LLOQ |
|  | AW4M1 |  |  | NS |  | <LLOQ |  | <LLOQ |
|  | BF2F3 |  |  | NS |  | NS |  | <LLOQ |
|  | BJ3F3 |  |  | NS |  | <LLOQ |  | <LLOQ |
|  | BJ3M2 |  |  | NS |  | NS |  | <LLOQ |
|  | BK1M4 |  |  | NS |  | <LLOQ |  | <LLOQ |
|  | BK2F1 |  |  | NS |  | <LLOQ |  | <LLOQ |
|  | BK2M4 |  |  | NS |  | NS |  | <LLOQ |
|  | BM2F1 |  |  | <LLOQ |  | <LLOQ |  | <LLOQ |
|  | BM2F2 |  |  | NS |  | <LLOQ |  | <LLOQ |
|  | BM2M1 |  |  | <LLOQ |  | <LLOQ |  | <LLOQ |
| *ColQ-/-*  Mota | AW2M1 | 20 mg/kg at P22 followed by weekly 10 mg/kg | P29 | NS | P43 | NS | P66 | <LLOQ |
|  | AW2M2 |  |  | NS |  | NS |  | <LLOQ |
|  | BF2F1 |  |  | NS |  | NS |  | <LLOQ |
|  | BF2F2 |  |  | NS |  | <LLOQ |  | <LLOQ |
|  | BF2M3 |  |  | NS |  | NS |  | <LLOQ |
|  | BJ3F4 |  |  | NS |  | NS |  | <LLOQ |
|  | BK1M2 |  |  | NS |  | NS |  | <LLOQ |
|  | BK1M3 |  |  | NS |  | NS |  | <LLOQ |
|  | BK2F2 |  |  | NS |  | NS |  | <LLOQ |
|  | BX4F3 |  |  | NS |  | NS |  | <LLOQ |
| *ColQ-/-*  3B2 | AV2F1 | 20 mg/kg at P22 followed by weekly 10 mg/kg | P29 | NS | P43 | NS | P66 | 83.8 |
|  | AW4F4 |  |  | NS |  | NS |  | 15.3 |
|  | AW4M2 |  |  | NS |  | NS |  | 39.3 |
|  | AW4M4 |  |  | NS |  | 12.5 |  | 32.1 |
|  | BI1F2 |  |  | NS |  | <LLOQ |  | 88.4 |
|  | BJ1F3 |  |  | NS |  | NS |  | 91.9 |
|  | BJ1M4 |  |  | NS |  | NS |  | 70.8 |
|  | BJ2F3 |  |  | NS |  | NS |  | 86.5 |
|  | BJ4F2 |  |  | NS |  | NS |  | 94.8 |
|  | BJ4M2 |  |  | NS |  | 37.4 |  | 108.6 |
|  | BK2M2 |  |  | NS |  | NS |  | 96.7 |
|  | BK3M6 |  |  | NS |  | NS |  | 128.6 |
|  | BX2M1 |  |  | NS |  | NS |  | 138.6 |

Levels of circulating 3B2 were below the levels of detection in the WT and *ColQ*^-/-^ (Mot) animals. While circulating 3B2 was clearly detectible in the *ColQ*^-/-^ (3B2) animals, due to the lack of serum available at P43, we were unable to tell if there had been an accumulation. However, the two animals for which we had adequate samples at both P43 and P66, showed a more than 100% increase in circulating 3B2. The levels observed at the end of the study in the *ColQ*^-/-^ mice were considerably higher than those in the *Agrn^nmf380^* mice, suggesting the lack of changes observed in the *ColQ*^-/-^ (3B2) animals was not attributable to underdosing. **NS** no samples taken (Animal care guidelines dictate a minimum body weight that must be reached before blood collection can be performed), **LLOQ** Lower limit of quantification (3B2 0.1µg/mL), **NA** not applicable. **NES** not enough sample.

**Supplementary Table 4: Post hoc analysis of inverted hanging wire test.**

|  | **Non-holder** | **Holder** |
| --- | --- | --- |
| **WT** | 0 | 6 |
| ***Agrn^nmf380^* (Mot)** | 3 | 0 |
| ***Agrn^nmf380^*(3B2)** | 0 | 6 |

Initial analysis of inverted hanging wire test showed no significant differences in motor performance between *Agrn^nmf380^* (Mot) mice and *Agrn^nmf380^* (3B2) mice. Mice were classified as non-holders (<2 seconds) and holders (> 2 seconds). Fisher’s exact test was performed and showed there was a significant improvement in treated animals at P31, p=0.0022. n=6.

**Supplementary Table 5: Morphological variables of NMJs from *Agrn^nmf380^* mice**.

| **Variable** | **WT (7)** | ***Agrn^nmf380^* Mot (3)** | ***Agrn^nmf380^* 3B2 (5)** |
| --- | --- | --- | --- |
| **Core Variables** |  |  |  |
| **Presynaptic** |  |  |  |
| Absent Synaptophysin staining | 0 | 0 | 0 |
| Nerve terminal area (µm^2^) | 184.3±69.5 | 105.3±92.9 | 167.1±110.8 |
| Nerve terminal perimeter (µm) | 216.6±65.8 | 147±105.2 | 282.8±176.2 |
| Number of terminal branches | 27.0±13.3 | 19.5±9.8 | 49.0±63.0 |
| Number of branch points | 15.8±7.8 | 8.4±6.4 * | 9.6±8.1 * |
| Total length branches (µm) | 104.3±33.1 | 67.0±48.5 | 114.2±73.1 |
| **Postsynaptic** |  |  |  |
| Absent AChR staining (%) | 17.7±11.9 | 52.8±47.4 | 18.6±16.8 |
| AChR area (µm^2^) | 134.0±96.0 | 21.0±45.6 **** | 57.3±56.2 *** |
| AChR perimeter (µm) | 200.3±125.7 | 50.4±100.7 ** | 165.9±145.2 # |
| Endplate area (µm^2^) | 317.9±203.4 | 70.6±116.9 | 478.1±475.9 ## |
| Endplate perimeter (µm) | 74.7±37.2 | 26.2±43.2 | 113.1±81.8 ## |
| Endplate diameter (µm) | 33.1±26.0 | 15.2±26.2 | 45.6±31.4 # |
| **Derived Variables** |  |  |  |
| **Presynaptic** |  |  |  |
| Average length of branches (µm) | 4.5±2.0 | 3.8±1.8 | 2.9±1.1 * |
| Complexity | 4.5±0.5 | 3.7±0.9 * | 4.4±0.9 |
| **Postsynaptic** |  |  |  |
| Average area of AChR clusters | 131.5±97.9 | 48.2±70.6 | 11.2±10.7 *** |
| Fragmentation | 0.2±0.3 | 0.6±0.4 * | 0.8±0.1 **** |
| Compactness (%) | 42.5±14.0 | 29.1±18.5 | 14.2±10.6 **** |
| Overlap (%) | 64.6±15.3 | 11.9±24.2 **** | 11.9±14.3 **** |
| Area of synaptic contact (µm^2^) | 101.4±51.7(60) | 8.2±23.8 **** | 10.8±14.8 **** |
| **Associated Nerve Variables** |  |  |  |
| Axon diameter (µm) | 2.1±0.8 | 1.7±0.5 | 2.1±0.7 |
| Number of axonal inputs | 1.0±0.1 | 1.1±0.3 | 1.0±0.1 |

Initial analysis of NMJ structure in the soleus revealed no sex related differences in variables and therefore data has been combined for males and females. Analysis was performed using previously published method.^29^ Data presented as mean ± s.d.. Nested 1-way ANOVA with Tukey’s multiple comparisons correction. WT n=7, *Agrn^nmf380^* (Mot) n=3, *Agrn^nmf380^* (3B2) n=5. An average of 9.93 NMJs were analysed per mouse.

^*^Significantly different from WT, ^#^significantly different from Mot. ^*/#^P<0.05, ^**/##^P<0.005, ^***/###^P<0.001, ^****/####^P<0.0001.

**Supplementary Table 6: Morphological variables of NMJs from WT and *ColQ^-/-^* mice.**

| **Variable** | **WT (6)** | ***ColQ^-/-^* (Mot) (6)** | ***ColQ^-/-^* (3B2) (6)** |
| --- | --- | --- | --- |
| **Core Variables** |  |  |  |
| **Presynaptic** |  |  |  |
| Absent Synaptophysin staining | 0 | 0 | 0 |
| Nerve terminal area (µm^2^) | 154.3±76.7 | 49.8±35.3 **** | 28.7±18.7 ****/# |
| Nerve terminal perimeter (µm) | 168.6±80.9 | 70.2±42.9 **** | 44.3±27.9 **** |
| Number of terminal branches | 19.3±11.8 | 12.3±8.3 * | 8.2±5.5 ** |
| Number of branch points | 12.3±7.7 | 3.5±2.9 **** | 1.9±1.9 **** |
| Total length branches (µm) | 77.5±42.0 | 26.2±17.3 **** | 16.3±11.3 **** |
| **Postsynaptic** |  |  |  |
| Absent AChR staining (%) | 0 | 0 | 0 |
| AChR area (µm^2^) | 160.4±84.8 | 64.6±38.5 **** | 33.4±29.4 ****/# |
| AChR perimeter (µm) | 174.6±91.4 | 97.2±53.3 *** | 64.8±43.9 **** |
| Endplate area (µm^2^) | 315.0±167.3 | 154.8±99.4 *** | 102.8±99.0 **** |
| Endplate perimeter (µm) | 79.2±22.6 | 57.3±22.5 ** | 45.2±21.9 **** |
| Endplate diameter (µm) | 32.7±16.7 | 22.7±12.7 ** | 17.7±11.1 *** |
| **Derived Variables** |  |  |  |
| **Presynaptic** |  |  |  |
| Average length of branches (µm) | 4.7±3.3 | 2.3±1.2 **** | 2.3±1.4 **** |
| Complexity | 4.0±0.8 | 2.9±0.8 **** | 2.4±0.7 ****/# |
| **Postsynaptic** |  |  |  |
| Average area of AChR clusters | 117.4±74.0 | 27.0±25.1 **** | 10.0±11.5 ****/# |
| Fragmentation | 0.2±0.3 | 0.5±0.3 ** | 0.7±0.3 *** |
| Compactness (%) | 52.5±12.9 | 44.9±12.5 | 34.3±12.1 ***/# |
| Overlap (%) | 63.2±14.8(122) | 38.3±15.0 **** | 35.4±18.6 **** |
| Area of synaptic contact (µm^2^) | 102.5±57.6 | 24.6±17.5 **** | 11.5±9.8 **** |
| **Associated Nerve Variables** |  |  |  |
| Axon diameter (µm) | 2.4±1.1 | 1.4±0.7 ** | 1.3±0.7 *** |
| Number of axonal inputs | 1.0±0.1 | 1.0±0.3 | 1.0±0.1 |

Initial analysis of NMJ structure in the soleus revealed no sex related differences in variables and therefore data has been combined for males and females. Analysis was performed using previously published method. ^29^ Data presented as mean ± s.d.. Nested 1-way ANOVA with Tukey’s multiple comparisons correction. n=6 animals per group. An average of 20.72 NMJs were analysed per mouse.

^*^Significantly different from WT, ^#^significantly different from Mot. ^*/#^P<0.05, ^**/##^P<0.005, ^***/###^P<0.001, ^****/####^P<0.0001.
